# Supplementary material for: Prediction of causal genes at GWAS loci with pleiotropic gene regulatory effects using sets of correlated instrumental variables
Source: PLoS Genet. 2024 Nov 11;20(11):e1011473. doi: 10.1371/journal.pgen.1011473 (PMC11581411; doi:10.1371/journal.pgen.1011473)
Supplement: S1 File — Contains 15 pages of supplementary methods. (PDF) [file pgen.1011473.s001.pdf]

Supplementary Methods

Prediction of causal genes at GWAS loci with pleiotropic gene regulatory effects using sets of correlated instrumental variables

Mariyam Khan[1], Adriaan-Alexander Ludl[1], Sean Bankier[1], Johan L.M. Björkegren[2,3], Tom Michoel[1,*]

[1] Computational Biology Unit, Department of Informatics, University of Bergen, PO Box 7803, 5020 Bergen, Norway

[2] Department of Medicine, (Huddinge), Karolinska Institutet, 141 57 Huddinge, Sweden

[3] Department of Genetics & Genomic Sciences, Institute of Genomics and Multiscale Biology, Icahn School of Medicine at Mount Sinai, New York, NY, 10029-6574, USA

[*] Corresponding author, email: tom.michoel@uib.no

S1 Instrumental sets and the method of path coefficients

To illustrate Brito & Pearl's instrumental set condition and the method of path coefficients for multivariate Mendelian randomization (MVMR), we consider the simplest causal diagrams with one and two exposures (Fig. 1A-C in the paper), this time with the structural equation model (SEM) coefficients labelling the edges:

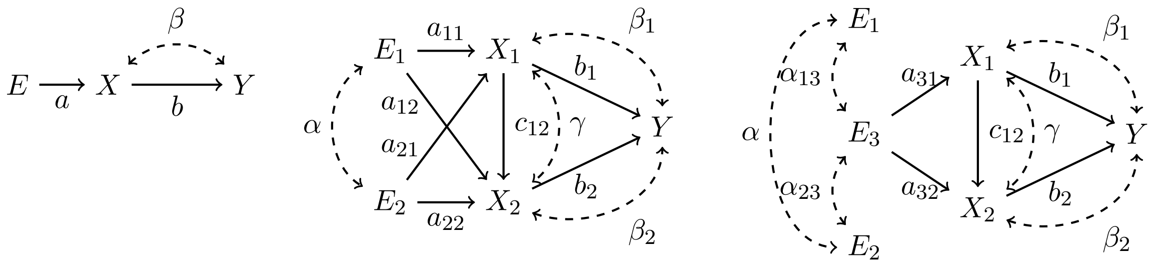

We also show the graphs obtained after removing the edges between the exposures and outcome, which appear in Brito & Pearl's instrumental set condition:

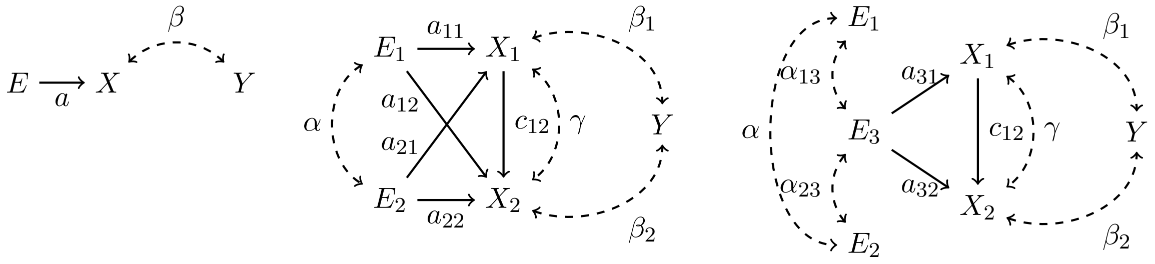

S1.1 Univariate MR

The method of path coefficients states that the covariance between a pair of variables equals $\sum_p T(p)$, where $T(p)$ is the product of the SEM paramaters of the edges along the path and the summation ranges over unblocked paths p connecting the two variables. A path is unblocked if it does not contain a collider, that is, a pair of consecutive edges pointing at the common node.

For the pair (E, X), there is one connecting path, $E \to X$ with path coefficient a, hence $\sigma_{EX} = a$.

For the pair (E, Y), there are two connecting paths, $E \to X \to Y$ with path coefficient ab, and $E \to X \leftrightarrow Y$, which is blocked by the collider at X. Hence only the first path contributes and $\sigma_{EY} = ab$.

These two path coefficients are sufficient to solve for the causal parameter of $X \to Y$:

$$b = \frac{\sigma_{EY}}{\sigma_{EX}}$$

The same result can of course be derived by manipulating the corresponding SEM:

$$E = U_E$$
$$X = aE + U_X$$
$$Y = bX + U_Y$$

with U_E, U_X, U_Y noise variables with covariances $\mathrm{Cov}(U_E, U_X) = \mathrm{Cov}(U_E, U_Y) = 0$. Assuming standardized variables, these imply immediately that $\mathrm{Cov}(X, E) = a\,\mathrm{Var}(E) = a$. Substituting this result in the equation for Y gives $\mathrm{Cov}(Y, E) = b\,\mathrm{Cov}(X, E) = ba$, or $b = \mathrm{Cov}(Y, E)/\,\mathrm{Cov}(X, E) = \sigma_{EY}/\sigma_{EX}$.

We can also verify that the singleton set $\{E\}$ is an instrumental set for Y relative to X. Take the path $p = E \to X \to Y$ and the pair (E, p). Then

1. E is a non-descendant of Y and p is an unblocked path between E and Y containing X.
2. E is d-separated from Y in the graph where the edge $X \to Y$ is removed, because in the truncated graph, the only path between E and Y is $E \to X \leftrightarrow Y$, which is blocked by the collider at X.
3. Condition 3 is moot because there the instrumental set is a singleton set.

S1.2 Two-variate MR

First we apply the method of path coefficients. We note that Y is d-separated from E_1 and E_2 in the graph $\overline{\mathcal{G}}$ which is obtained from the causal graph $\mathcal{G}$ by deleting the directed edges $X_1 \to Y$ and $X_2 \to Y$. It follows that all d-connected paths between E_1 or E_2 and Y in $\mathcal{G}$ must have either $X_1 \to Y$ or $X_2 \to Y$ as their final edge. In other words, in the sum $\sum_p T(p)$ over unblocked paths between E_i and Y, no path appears that contains the bidirectional edge $X_i \leftrightarrow Y$, because such paths will necessarily have a collider at X_i. By the method of path coefficients, it follows that the covariances σ_{E_1Y} and σ_{E_2Y} are linear in b_1 and b_2 without constant terms:

$$\sigma_{E_1Y} = R_{11}b_1 + R_{12}b_2$$
$$\sigma_{E_2Y} = R_{21}b_1 + R_{22}b_2$$

Because *(i)* every d-connected path between E_i and Y that has $X_j \to Y$ as its terminal edge must have X_j as its next to terminal vertex, *(ii)* every such path can be truncated to a d-connected subpath from E_i to X_j, and *(iii)* every d-connected path from E_i to X_j can be extended by the edge $X_j \to Y$ into a d-connected path between E_i and Y, it follows that the coefficients R_{ij} must be equal to the covariances $\sigma_{E_iX_j}$, and hence

$$\sigma_{E_1Y} = \sigma_{E_1X_1}b_1 + \sigma_{E_1X_2}b_2 \tag{S1}$$
$$\sigma_{E_2Y} = \sigma_{E_2X_1}b_1 + \sigma_{E_2X_2}b_2. \tag{S2}$$

We can write this set of equations in matrix notation as

$$\Sigma_{EX} b = \Sigma_{EY}, \tag{S3}$$

where $\Sigma_{EX} = (\sigma_{E_i X_j})$, $b = (b_1, b_2)^T$, and $\Sigma_{EY} = (\sigma_{E_2 Y}, \sigma_{E_2 Y})^T$. Hence

$$b = \Sigma_{EX}^{+} \Sigma_{EY} = (\Sigma_{EX}^{T} \Sigma_{EX})^{-1} \Sigma_{EX}^{T} \Sigma_{EY}. \tag{S4}$$

For the case of two genes and two variants, we can solve eq. (S5) explicitly:

$$\Sigma_{EX}^{+} = \frac{1}{\sigma_{E_1 X_1} \sigma_{E_2 X_2} - \sigma_{E_1 X_2} \sigma_{E_2 X_1}} \begin{pmatrix} \sigma_{E_2 X_2} & -\sigma_{E_1 X_2} \\ -\sigma_{E_2 X_1} & \sigma_{E_1 X_1} \end{pmatrix}$$

and hence

$$b_1 = \frac{\sigma_{E_1 Y} \sigma_{E_2 X_2} - \sigma_{E_2 Y} \sigma_{E_1 X_2}}{\sigma_{E_1 X_1} \sigma_{E_2 X_2} - \sigma_{E_2 X_1} \sigma_{E_1 X_2}} \tag{S5}$$

$$b_2 = \frac{\sigma_{E_2 Y} \sigma_{E_1 X_1} - \sigma_{E_1 Y} \sigma_{E_2 X_1}}{\sigma_{E_1 X_1} \sigma_{E_2 X_2} - \sigma_{E_1 X_2} \sigma_{E_2 X_1}} \tag{S6}$$

We see immediately that if E_1 and E_2 are independent, X_1 and X_2 are independent, and E_1 and E_2 are instrumental variables for X_1 and X_2 respectively (that is, we have two independent univariate instrumental variable graphs pointing at Y), then we recover the univariate instrumental variable estimates $b_i = \sigma_{E_i Y} / \sigma_{E_i X}$. We also see that as the correlation α between E_1 and E_2 tends to one (that is, E_1 and E_2 become identical), then the expressions for b_1 and b_2 become undefined (0/0).

The same result can also be derived by manipulation of the SEM:

$$\begin{aligned} E_1 &= U_{E_1} \\ E_2 &= U_{E_2} \\ X_1 &= a_{11} E_1 + a_{12} E_2 + U_{X_1} \\ X_2 &= a_{12} E_1 + a_{22} E_2 + c_{12} X_1 + U_{X_2} \\ Y &= b_1 X_1 + b_2 X_2 + U_Y \end{aligned}$$

with noise variables being pairwise independent if there is no bidirected edge in the causal graph. Assuming standardized variables, we get immediately

$$\begin{aligned} \mathrm{Cov}(Y, E_1) &= b_1 \,\mathrm{Cov}(X_1, E_1) + b_2 \,\mathrm{Cov}(X_2, E_1) \\ \mathrm{Cov}(Y, E_2) &= b_1 \,\mathrm{Cov}(X_1, E_2) + b_2 \,\mathrm{Cov}(X_2, E_2) \end{aligned}$$

which again leads to the equation

$$\Sigma_{EX} b = \Sigma_{EY}$$

We now verify that the set $\{E_1, E_2\}$ is an instrumental variable set for Y relative to $\{X_1, X_2\}$. Define the paths $p_1 = E_1 \to X_1 \to Y$ and $p_2 = E_2 \to X_2 \to Y$.

1. Each E_i is a non-descendant of Y and p_i is a path between E_i and Y including the edge $X_i \to Y$.
2. Each E_i is d-separated from Y in the graph $\overline{\mathcal{G}}$ obtained by deleting the directed edges $X_1 \to Y$ and $X_2 \to Y$, as was already noted before.
3. E_2 does not appear in the path p_1 and the paths p_1 and p_2 have no common variable.

To clarify the third instrumental set condition, we consider an example graph where condition 3 is *not* satisfied despite conditions 1 and 2 being satisfied is given in Figure 1C (shown here is a simplified version without edges connecting X_1 and X_2):

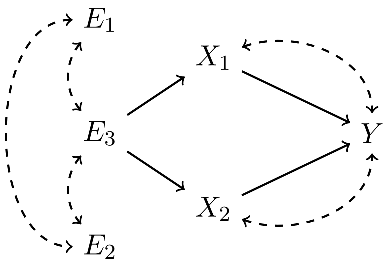

In this case, E_1 and E_2 are merely correlated with a single causal variant E_3. In this case all paths between the instruments and outcome Y pass through E_3. Intuitively, E_1 and E_2 then provide redundant information and are insufficient to determine b_1 and b_2. If we use E_1 and E_2 as potential instruments with paths $p_1 = E_1 \leftrightarrow E_3 \to X \to Y$ and $p_2 = E_2 \leftrightarrow E_3 \to X \to Y$, we see that both paths have the common variable $V = E_3$. Since the truncation $p_1[E_1 \sim E_3] = E_1 \leftrightarrow E_3$ points *towards* the common variable E_3, and the truncation $p_2[E_3 \sim Y] = E_3 \to X_2 \to Y$ points *away from* the common variable E_3, condition 3 is *not* satisfied. The same result is easily obtained using any other combination of instruments from $\{E_1, E_2, E_3\}$ or any other labelling of the paths as p_1 or p_2.

An example graph where condition 3 is satisfied with paths having a common variable is given by Brito & Pearl:

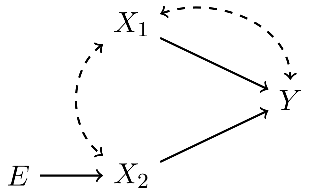

Here E acts as an instrument for X_2 and X_2 as an instrument for X_2, that is, the instrument set is $\{X_2, E\}$. Using the paths $p_1 = X_2 \leftrightarrow X_1 \to Y$ and $p_2 = E \to X_2 \to Y$, we can verify that the three instrumental set conditions are satisfied. In particular, for the third condition, we have the common variable X_2, and both $p_1[E \sim X_2] = E \to X_2$ and $p_2[X_2 \to Y] = X_2 \leftrightarrow X_1 \to Y$ point to X_2. The example is of course artificial in that it requires the absence of a bidirected edge $X_2 \leftrightarrow Y$.

In conclusion, while standard covariance algebra using the SEM equations easily leads to the same answer as the method of path coefficients in these simple examples, the power of Brito

& Pearl's instrumental set condition lies in the generality of the result for any number of exposures and any kind of causal or confounding relations between them.

S2 Standard error calculations for causal effect estimators

S2.1 Variance of the GMM estimator

The GMM causal effect estimator $\hat{c}_{GMM}$ can be written as

$$\hat{c}_{GMM} = ((X^T E) \cdot (E^T E)^{-1} \cdot (E^T X))^{-1} (X^T E) \cdot (E^T E)^{-1} \cdot E^T y$$

Let

$$((X^T E) \cdot (E^T E)^{-1} \cdot (E^T X))^{-1} (X^T E) \cdot (E^T E)^{-1} \cdot E^T = A$$

Then using the rule $\mathrm{Var}(Ay) = A\,\mathrm{Var}(y) A^T$ for a matrix A and a vector y, we get

$$\begin{aligned}\mathrm{Var}(\hat{c}_{GMM}) = ((X^T E) \cdot (E^T E)^{-1} \cdot (E^T X))^{-1} (X^T E) \cdot (E^T E)^{-1} \cdot E^T\,\mathrm{Var}(y) E (E^T E)^{-1} \\ (E^T X)\big(((X^T E) \cdot (E^T E)^{-1} \cdot (E^T X))^{-1}\big)^T\end{aligned}$$

$$\begin{aligned}\mathrm{Var}(\hat{c}_{GMM}) = \mathrm{Var}(y)((X^T E) \cdot (E^T E)^{-1} \cdot (E^T X))^{-1} (X^T E) \cdot (E^T E)^{-1} \cdot E^T E (E^T E)^{-1} \\ (E^T X)((X^T E) \cdot (E^T E)^{-1} \cdot (E^T X))^{-1}\end{aligned}$$

If $((X^T E) \cdot (E^T E)^{-1} \cdot (E^T X)) = P$, and using the fact that $E^T\,\mathrm{Var}(y) E (E^T E)^{-1} = \mathrm{Var}(y) I$, we have

$$\mathrm{Var}(\hat{c}_{GMM}) = \mathrm{Var}(y) P^{-1} P P^{-1}$$

Hence,

$$\mathrm{Var}(\hat{c}_{GMM}) = \mathrm{Var}(y)((X^T E) \cdot (E^T E)^{-1} \cdot (E^T X))^{-1}$$

S2.2 Variance of the least-squares estimator

The least-squares causal effect estimator $\hat{c}_{LS}$ can be written as

$$\hat{c}_{LS} = ((X^T E) \cdot (E^T X))^{-1} (X^T E) \cdot E^T y$$

Repeating the calculation above, we again find

$$\mathrm{Var}(\hat{c}_{LS}) = \mathrm{Var}(y)\big((X^T E) \cdot (E^T E)^{-1} (E^T X)\big)^{-1}$$

S2.3 Standard errors

As we saw before, both the GMM and LS estimators have the variance given by:

$$\text{Var}(\hat{c}) = \text{Var}(y)(X^T E(E^T E)^{-1} E^T X)^{-1}$$

and this formula applies in both the over-determined and just-determined cases.

$\hat{c}$ is asymptotically distributed as $\hat{c} \xrightarrow{d} \mathcal{N}(c, \text{Var}(\hat{c}))$, where c is the true population parameter. Hence by the central limit theorem,

$$\sqrt{n}(\hat{c} - c) \xrightarrow{d} \mathcal{N}\big(0, \hat{\sigma}^2(X^T E(E^T E)^{-1} E^T X)^{-1}\big)$$

where $\hat{\sigma}^2 = \text{Var}(y)$ and is approximated using $\hat{\sigma}^2 \approx \frac{1}{n}(y - \hat{X}\hat{c})^T(y - \hat{X}\hat{c})$. This covariance estimator for heteroscedastic errors requires individual level data and hence not suitable for summary statistics-based MR estimation.

When we do not have access to individual level data, we approximate $\text{Var}(y)$ using the argument made in [**24**], where the authors explain that $\hat{\sigma}^2$ represents the variance of the outcome trait unexplained by the causal predictor, which in reality is very close to 1. Hence for the calculation of standard errors, we always use

$$se(\hat{c}_i) = \frac{\omega_i}{\sqrt{n}}$$

where

$$\omega_i^2 = \big[(X^T E(E^T E)^{-1} E^T X)^{-1}\big]_{ii}$$

For the sample size n, using the outcome dataset's sample size for the standard error calculation ensures that we account for the variability in the outcome variable. In our application to coronary artery disease, for the study ebi-a-GCST003116, we have a sample size of 141,217 individuals which was used for all standard error calculations.

For the estimate, p-value is calculated as:

$$\text{p-value} = 2 \cdot \Phi(-|\hat{c}/se(\hat{c})|) \tag{S7}$$

where Φ is the cumulative distribution function (CDF) of the standard normal distribution.

S2.4 Standard errors for causal estimates using bootstrapping methods.

In addition to approximating standard errors from one-sample estimation, we have employed bootstrapping methods to estimate the standard errors of our causal estimates as follows.

Consider a model with $k = 2$ genes ($X = (X_1, X_2)$) and $p = 2$ SNPs serving as instruments ($E = (E_1, E_2)$) that influence an outcome variable Y. Assume the following genetic association estimates are available:

- $\hat{\mathbf{b}} = [\hat{b}_{ij}]$: A 2×2 matrix of estimated genetic associations between SNPs (E_i) and gene expressions (X_j).
- $\mathbf{SE}_X = [se_{\hat{b}_{ij}}]$: A 2×2 matrix of standard errors corresponding to $\hat{\mathbf{b}}$.
- $\hat{\mathbf{a}} = \begin{bmatrix} \hat{a}_1 \\ \hat{a}_2 \end{bmatrix}$: A vector of length 2 representing the estimated genetic associations between SNPs (E) and the outcome Y.
- $\mathbf{SE}_Y = \begin{bmatrix} se_{\hat{a}_1} \\ se_{\hat{a}_2} \end{bmatrix}$: A vector of standard errors for $\hat{\mathbf{a}}$, also of length 2.

We aim to generate N bootstrap samples of $\hat{\mathbf{a}}$ and $\hat{\mathbf{b}}$ to estimate the standard errors of causal effects. We use two regression models: one from a GWAS study, which provides genetic association estimates between the SNPs (E) and the outcome variable Y, and another from an eQTL study, which provides genetic association estimates between the SNPs (E) and the gene expressions X.

First, we derive an expression for the variance-covariance matrix of $\hat{\mathbf{a}}$ i.e. $\hat{a}_1$ and $\hat{a}_2$, estimated using separate regressions.

The two linear regression models are as follows:

First Regression:

$$Y = E_1 a_1 + \epsilon_1$$

Second Regression:

$$Y = E_2 a_2 + \epsilon_2$$

Where:

- Y is an $N \times 1$ vector of the dependent variable.
- E_1 and E_2 are $N \times 1$ vectors of regressors.
- a_1 and a_2 are scalars to be estimated.
- ϵ_1 and ϵ_2 are $N \times 1$ vectors of error terms.

Assumptions:

$$\mathbb{E}[\epsilon_1] = \mathbb{E}[\epsilon_2] = 0, \quad \mathrm{Var}(\epsilon_1) = \sigma_1^2 I_N, \quad \mathrm{Var}(\epsilon_2) = \sigma_2^2 I_N, \quad \mathrm{Cov}(\epsilon_1, \epsilon_2) = 0$$

The Ordinary Least Squares (OLS) estimators for a_1 and a_2 are obtained by minimizing the sum of squared residuals and we grt:

Estimator for a_1:

$$\hat{a}_1 = (E_1^\top E_1)^{-1} E_1^\top Y$$

Estimator for a_2:

$$\hat{a}_2 = (E_2^\top E_2)^{-1} E_2^\top Y$$

If all variables are standardized, then the covariance between $\hat{a}_1$ and $\hat{a}_2$ can be expressed as:

$$\mathrm{Cov}\begin{pmatrix}\hat{a}_1\\ \hat{a}_2\end{pmatrix} = \begin{bmatrix}\sigma_1^2 & 0\\ 0 & \sigma_2^2\end{bmatrix} \tag{S8}$$

For two SNPs E and the outcome Y, we can hence sample bootstrapped genetic associations $\mathbf{a}^{boot}$ using a bivariate normal distribution:

$$\mathbf{a}^{boot} \sim \mathcal{N}(\mathbf{a}, \Sigma_{\mathbf{a}})$$

where $\Sigma_{\mathbf{a}}$ is constructed from the standard errors as given in S8 and σ_1^2 and σ_1^2 are given by $\mathrm{se}(\hat{a_1})^2$ and $\mathrm{se}(\hat{a_2})^2$, respectively.

Similarly for genes, we would have four linear regression models:

- **First Regression:**
$$X_1 = b_{11}E_1 + \epsilon_1$$
- **Second Regression:**
$$X_1 = b_{12}E_2 + \epsilon_2$$
- **Third Regression:**
$$X_2 = b_{21}E_1 + \epsilon_3$$
- **Fourth Regression:**
$$X_2 = b_{22}E_2 + \epsilon_4$$

Here we assume

- E_1 and E_2 are independent.
- X_1 and X_2 are independent conditional on E_1 and E_2.
- The error terms have the following properties:

$$\mathbb{E}[\epsilon_i] = 0 \quad \text{for } i = 1, 2, 3, 4,$$
$$\mathrm{Var}(\epsilon_i) = \sigma_i^2 I_N,$$
$$\mathrm{Cov}(\epsilon_i, \epsilon_j) = 0 \quad \text{if the errors are from different dependent variables,}$$
$$\text{i.e., when } i \text{ and } j \text{ refer to different } X.$$
$$\mathrm{Cov}(\epsilon_1, \epsilon_2) = 0, \quad \mathrm{Cov}(\epsilon_3, \epsilon_4) = 0$$

For standardized variables, we will have:

$$\mathrm{Cov}\begin{pmatrix}\hat{b}_{11}\\ \hat{b}_{12}\\ \hat{b}_{21}\\ \hat{b}_{22}\end{pmatrix} = \begin{bmatrix}\sigma_1^2 & 0 & 0 & 0\\ 0 & \sigma_2^2 & 0 & 0\\ 0 & 0 & \sigma_3^2 & 0\\ 0 & 0 & 0 & \sigma_4^2\end{bmatrix} \tag{S9}$$

For SNPs E and the genes X, we can hence sample bootstrapped genetic associations $\mathbf{b}^{boot}$ using a multivariate normal distribution:

$$\mathbf{b}^{boot} \sim \mathcal{N}(\mathbf{b}, \Sigma_{\mathbf{b}})$$

where $\Sigma_{\mathbf{b}}$ is constructed from the standard errors as given in S9 and σ_1^2, σ_2^2, σ_3^2 and σ_4^2 are given by $\text{se}(\hat{b_{11}})^2$, $\text{se}(\hat{b_{12}})^2$, $\text{se}(\hat{b_{21}})^2$ and $\text{se}(\hat{b_{22}})^2$, respectively.

Finally this is the workflow

1. **Generate Bootstrapped Samples**:
   - For SNPs E and genes X, sample bootstrapped genetic associations $\mathbf{b}^{boot}$ using a multivariate normal distribution:

     $$\mathbf{b}^{boot} \sim \mathcal{N}(\mathbf{b}, \Sigma_{\mathbf{b}})$$

     where $\Sigma_{\mathbf{b}}$ is constructed from the standard errors and correlation matrix as given in S9.
   - For SNPs E and the outcome Y, we can hence sample bootstrapped genetic associations $\mathbf{a}^{boot}$ using a multivariate normal distribution:

     $$\mathbf{a}^{boot} \sim \mathcal{N}(\mathbf{a}, \Sigma_{\mathbf{a}})$$

     where $\Sigma_{\mathbf{a}}$ is constructed from the standard errors and correlation matrix as given in S8
2. **Estimate Causal Effects**:
   - For each bootstrap sample, perform least squares (LS) estimation to compute:

     $$\hat{\theta}_{boot} = ((\mathbf{a}^{boot})^\top \mathbf{a}^{boot})^{-1} (\mathbf{a}^{boot})^\top \mathbf{b}^{boot})$$

   - For each bootstrap sample, perform GMM estimation to compute:

     $$\hat{\theta}_{boot} = ((\mathbf{a}^{boot})^\top {\Sigma_E}^{-1} \mathbf{a}^{boot})^{-1} (\mathbf{a}^{boot})^\top {\Sigma_E}^{-1} \mathbf{b}^{boot})$$

     where Σ_E is the LD-matrix of SNPs
3. **Compute Standard Errors**:
   - Calculate the standard error of each causal effect estimate as the standard deviation of the bootstrap estimates performed for N repetitions:

     $$\text{SE}(\hat{\theta}_{boot}) = \sqrt{\frac{1}{N-1} \sum_{i=1}^{N} (\hat{\theta}_{boot,i} - \bar{\theta}_{boot})^2}$$

S3 Simulations

S3.1 Simulation of discrete LD-matrices

The formula for the linkage disequilibrium coefficient D_{ij}, where alleles at the first locus are denoted as $A_i \in \{0,1\}$ and alleles at the second locus are denoted as $B_j \in \{0,1\}$, is given by:

$$D_{ij} = P_{A_iB_j} - P_{A_i} \cdot P_{B_j}$$

- D_{ij} is the measure of linkage disequilibrium for the allele combination A_iB_j.
- $P_{A_iB_j}$ is the observed frequency of the haplotype combining allele A_i at one locus with allele B_j at the second locus.
- P_{A_i} is the frequency of allele A_i in the population.
- P_{B_j} is the frequency of allele B_j in the population.

The normalized value is given by $r_{ij} = \frac{D_{ij}}{\sqrt{P_{A_1} \cdot P_{A_2} \cdot P_{B_1} \cdot P_{B_2}}}$. Let us denote the denominator by P_a. Assume alleles A, B at two successive loci with the following initial allele frequencies:

- Locus A: $P(A_1) = p$, $P(A_2) = 1 - p$
- Locus B: $P(B_1) = q$, $P(B_2) = 1 - q$

Note that using the R function *ld_matrix* (all LD values are with respect to the major alleles in the 1000G dataset), we are given r_{11} where (A_1) and (B_1) are the major alleles at loci A and B. Hence we can calculate r_{ij} where $i \in \{0,1\}$ and $j \in \{0,1\}$.
To calculate the probabilities of transitioning from each allele at Locus A to each allele at Locus B, we start with the haplotype frequencies using D:

$$P(A_iB_j) = P(A_i) \times P(B_j) + r_{ij} \times P_a$$

For all A_iB_j where $i \in \{0,1\}$ and $j \in \{0,1\}$, frequencies can be calculated similarly, ensuring they sum to 1.

Transition probabilities are derived as follows:

$$P(B_j|A_i) = \frac{P(A_iB_j)}{P(A_i)}$$

Haplotype is a combination of alleles from different loci on the same chromosome. We first generate genotype data where we start with the allele frequencies at the first locus and use the transition probabilities to determine the allele at the next locus and so on. We do this twice and add the two vectors to get the haplotype data.

An example of a 4x4 true reference LD matrix, for four eQTLs (rs9457995, rs79390162, rs624249, rs56393506) using the R function *ld_matrix* is given by

$$\begin{bmatrix} 1 & -0.196 & 0.27 & -0.04 \\ -0.196 & 1 & 0.014 & -0.21 \\ 0.27 & 0.014 & 1 & -0.22 \\ -0.04 & -0.21 & -0.22 & 1 \end{bmatrix}$$

The simulated LD matrix of genotypes generated through our method is

$$\begin{bmatrix} 1 & -0.21 & -0.025 & 0.007 \\ -0.205 & 1 & 0.02 & 0.006 \\ -0.025 & 0.02 & 1 & -0.215 \\ 0.007 & 0.0067 & -0.21 & 1 \end{bmatrix}$$

We observe that the LD between successive SNPs is simulated accurately, but LD between SNPs more than one step away deviates from the true value due to the Markovian assumption of our simulation method.

We compared results on data simulated using the Markovian model to simulations with normally distributed instruments with the true LD matrix as covariance matrix and observed no qualitative differences, Hence we concluded that our genotype simulation model generates sufficiently complex LD matrices to test MVMR methods with correlated instruments.

S3.2 Simulation of biased LD matrices

In our analysis of GWAS and eQTL datasets, the LD matrix is utilized solely for causal effect estimation via the GMM estimator. This matrix (reference LD matrix) is derived from the 1000G population using the R function `ld_matrix`, which does not necessarily represent the genetic backgrounds of the GWAS and eQTL populations exactly.

To test the influence of biased LD matrices on the causal effect estimates, we simulated an LD matrix using the Wishart distribution centred around the reference LD matrix, with 50 degrees of freedom to model deviation from the reference LD matrix in the eQTL and GWAS populations. Simulated datasets for genotypes, exposures, and outcomes are generated using this matrix. For causal effect estimation, the reference LD matrix serves as the covariance matrix in the GMM estimator, modeling the scenario where the eQTL and GWAS samples are from the same overall population (e.g. European).

For the simulation on the accuracy of the LD matrix, we generated datasets for the *SLC22A3-LPA-PLG* locus (6 : 161089307) in liver. Causal effects of the genes on the outcome variable Y were calculated to be $c_{SLC22A3} = 0.15$, $c_{LPA} = -0.05$ and $c_{PLG} = -0.27$ when seven instruments were used, and the same values were also used in the other simulations. We compared the situations where we keep only $L = 3$ (LD threshold of 0.25), $L = 4$ (LD threshold of 0.3) , $L = 5$ (LD threshold of 0.4), $L = 6$ (LD threshold of 0.5), and $L = 7$ instruments (LD threshold of 0.8). We performed simulations where the sample size varied between $500 - 300000$. For the instrument effect sizes, we assumed that only three eQTLs are causal for all genes (their effect sizes sampled from a uniform distribution within the range $0.7 - 1.0$) and as the number of eQTLs increased with the LD threshold, we assumed them to be in LD (as reflected by the real LD matrix) with these three causal eQTLs without themselves being causal for the outcome.

The true reference LD matrix for three eQTLs:

$$\begin{bmatrix} 1. & -0.04 & -0.21 \\ -0.04 & 1. & -0.196 \\ -0.21 & -0.196 & 1. \end{bmatrix}$$

An example of an LD matrix for eQTL data and GWAS data generated from a Wishart distribution with 50 degrees of freedom:

$$\begin{bmatrix} 1. & -0.12 & -0.18 \\ -0.12 & 1. & -0.2 \\ -0.18 & -0.24 & 1. \end{bmatrix}$$

S3.3 Calculation of Conditional F-Statistics and Cochran's Q-Statistic

To mitigate issues related to weak instrument bias and horizontal pleiotropy in the STARNET data analysis, we employ the *Conditional F-statistic* and *Cochran's Q-statistic.* These statistics are reported using the *Mendelian Randomization* package, which differentiates between weak and strong instruments using a threshold of 10 for the *Conditional F-statistic.* This analysis utilizes summary data for instrument-exposure and instrument-outcome associations, alongside the correlation matrix of genetic associations with exposures. We validated this approach by comparing it with the *Conditional F-statistic* outcomes from the MVMR package, which requires individual-level data. Given the similarity of results, we proceeded with the *Mendelian Randomization* package for our final analyses.

S3.4 Bias of Two Sample MR

In this section, we elaborate on the bias introduced in Two Sample MR, as compared to one sample MR. In order to do this, we simulate the simplest scenario in MR, with one instrument E which causally impacts one exposure X, $E \rightarrow X$, through the causal parameter a, which in turn impacts outcome variable Y, $X \rightarrow Y$, through the causal parameter c, all normally distributed with Gaussian errors. Parameter of interest is c given by:

$$c = \frac{\sigma_{EY}}{\sigma_{EX}}$$

In two sample MR, σ_{EY} and σ_{EX} are estimated using two non-overlapping samples - *Sample 1* and *Sample 2.* We therefore need to compare $c - \hat{c}_{ij}$ with $c - \hat{c}_{ii}$ where $i, j \in \{1, 2\}$ corresponding to the sample label and $i \neq j$. c being the true causal effect $X \rightarrow Y$ and $\hat{c}_{i,j}$ is the estimated causal effect when σ_{EY} from sample, *Sample i* and σ_{EX} is estimated from sample, *Sample j.* As can be seen from , $c - \hat{c}_{ii}$ and $c - \hat{c}_{ij}$ go to zero, as sample size increases, as expected. However, for any sufficiently small sample size, we see $c - \hat{c}_{ij}$ for $i \neq j$ is significantly away from zero while $c - \hat{c}_{ii}$ is not. In other words for sufficiently small sample sizes, Two Sample MR estimates demonstrate significant bias as compared to one Sample MR. Notably, one can also see that the bias is greater for smaller values of a, which is expected as a dictates the variance of σ_{EX} distribution.

S3.5 Explained variance of the first PC for two variables

For a dataset with two variables, with correlation coefficient r between these variables and given that the variables are standardized, the covariance matrix Σ is:

$$\Sigma = \begin{bmatrix} 1 & r \\ r & 1 \end{bmatrix}$$

The eigenvalues λ of this covariance matrix represent the variances explained by the principal components. These eigenvalues are found by solving the characteristic equation:

$$\det(\Sigma - \lambda I) = 0$$

This results in the following equation:

$$\begin{vmatrix} 1-\lambda & r \\ r & 1-\lambda \end{vmatrix} = 0$$

$$(1-\lambda)^2 - r^2 = 0$$

$$1 - \lambda = \pm r$$

So, the eigenvalues are $\lambda_1 = 1 + r$ and $\lambda_2 = 1 - r$.

The explained variance of the first principal component is given by the proportion of the total variance (sum of eigenvalues) explained by the largest eigenvalue, that is

$$\frac{\lambda_1}{\lambda_1 + \lambda_2} = \frac{1+r}{(1+r)+(1-r)} = \frac{1+r}{2}$$

S3.6 Calculation of Type 1 error and power

Calculation of Type 1 error

We start with the locus on Chromosome 15:79124475 in the MAM tissue where the two genes *ADAMTS7* and *CTSH* are shared among a 100 instruments. We simulate discrete SNPs from the real LD-matrix of 8 remaining SNPS after putting a threshold of 0.72 of maximum LD.

1. For the simulation of Type 1 error, we always simulate from a model where the causal effect of *ADAMTS7* $c_{ADAMTS7} = 0.0$ and causal effect of *CTSH* $c_{CTSH} = 0.2$. Type 1 error is calculated by the number of times we reject the null model given that the null model is true and $|\hat{c}_{GMM}| > c_{thresh}$, where $\hat{c}_{GMM}$ is the estimated causal effect for $c_{ADAMTS7}$ using GMM estimation.

2. For each threshold of $c_{thresh} \in [0.01, 0.2]$, simulate data 1000 times from $c_{ADAMTS7} = 0.0$, $c_{CTSH} = 0.2$ and do the following:

   - Generate covariance matrix between genes and SNPs ($E^T X$), CAD outcome and SNPs ($E^T Y$) and LD matrix between SNPs ($E^T E$) for a sample size of $n = 2000$.

- Compute $\hat{c}_{GMM}$, the GMM estimator for *ADAMTS7*.

$$\hat{c}_{GMM} = ((X^T E) \cdot (E^T E)^{-1} \cdot (E^T X))^{-1} (X^T E) \cdot (E^T E)^{-1} \cdot E^T y$$

- Compute the error covariance matrix:

$$error = ((X^T E) \cdot (E^T E)^{-1} \cdot (E^T X))^{-1}$$

- Calculate residuals: $residuals = (y - \hat{X}\hat{c}_{GMM})^T (y - \hat{X}\hat{c}_{GMM})$
- Estimate residual variance:

$$\hat{\sigma}^2 = \frac{1}{n-2} \cdot (\text{residuals}^T \cdot \text{residuals})$$

- Calculate standard error in the GMM estimator for *ADAMTS7*:

$$\text{standard error} = \frac{\sqrt{\text{diag}(\text{error} \cdot \hat{\sigma}^2)}}{\sqrt{n}}$$

- Compute t-statistic:

$$t\_\text{statistic} = \frac{\hat{c}_{GMM}}{\text{standard error}}$$

- Calculate p-values:

$$p\_\text{values} = 2 \cdot (1 - \Phi(|t\_\text{statistic}|))$$

3. Calculate Type I error rate by counting the number of times the null hypothesis is rejected for *ADAMTS7* for all c_{thresh} values and $|\hat{c}_{GMM}| > c_{thresh}$.

Calculation of Power

1. For the simulation of power, we always simulate from a model where the causal effect of *ADAMTS7* $c_{ADAMTS7} = c_{true} \in [0.01, 0.2]$ and causal effect of *CTSH* $c_{CTSH} = 0.2$. The aim is to compare the null model $c_{ADAMTS7} = 0.0$ against an alternate model $c_{ADAMTS7} = c_{true}$. Hence power is calculated by the number of times we reject the null model given that the alternate model is true.

2. For each value of $c_{ADAMTS7} = c_{true} \in [0.01, 0.2]$, simulate data 1000 times from $c_{ADAMTS7}$ and $c_{CTSH} = 0.2$ follow the same steps as in Type 1 error with these changes.

   - Compute t-statistic:

   $$t\_\text{statistic} = \frac{\hat{c}_{GMM}}{\text{standard error}}$$

   - Calculate p-values:

   $$p\_\text{values} = 2 \cdot (1 - \Phi(|t\_\text{statistic}|))$$

3. Calculate power by counting the number of times the null hypothesis is rejected for *ADAMTS7* for all c_{true} values.

S3.7 Comparison with other MVMR methods

For the simulation on comparison with new MVMR methods **MVMR-Median**, **MVMR-Lasso**, **MVMR-Robust** and **MVMR-cML**, we generated datasets for the *SLC22A3-LPA-PLG* locus (6 : 161089307) in liver. Causal effects of the genes on the outcome variable Y were calculated to be $c_{SLC22A3} = 0.15$, $c_{LPA} = -0.05$ and $c_{PLG} = -0.27$ with eight instruments which were used for estimation for all methods.

For **MVMR-Median**, **MVMR-Lasso** and **MVMR-Robust**, we supplied the effect sizes and standard errors as generated in the simulations. For **MVMR-cML** we did the same and in addition supplied the following

- ***K_vec***: This parameter represents the set of potentially invalid instruments to consider in the model. For our eight instruments model, we fixed this to be a range of 0 to 5 for identification purposes.
- ***rho_mat***: This was taken as the sample correlation matrix of exposures and outcome. Since our exposures were from sample samples, we provided the sample correlation matrix of exposures and for correlation of exposures with outcome we took 0 as they are taken from different samples.
- For sample size we provided $n = 600$, as per instructions it is smallest sample size of the two datasets.
