# Supplementary figures and images for: Prediction of causal genes at GWAS loci with pleiotropic gene regulatory effects using sets of correlated instrumental variables
Source: PLoS Genet. 2024 Nov 11;20(11):e1011473. doi: 10.1371/journal.pgen.1011473 (PMC11581411; doi:10.1371/journal.pgen.1011473)

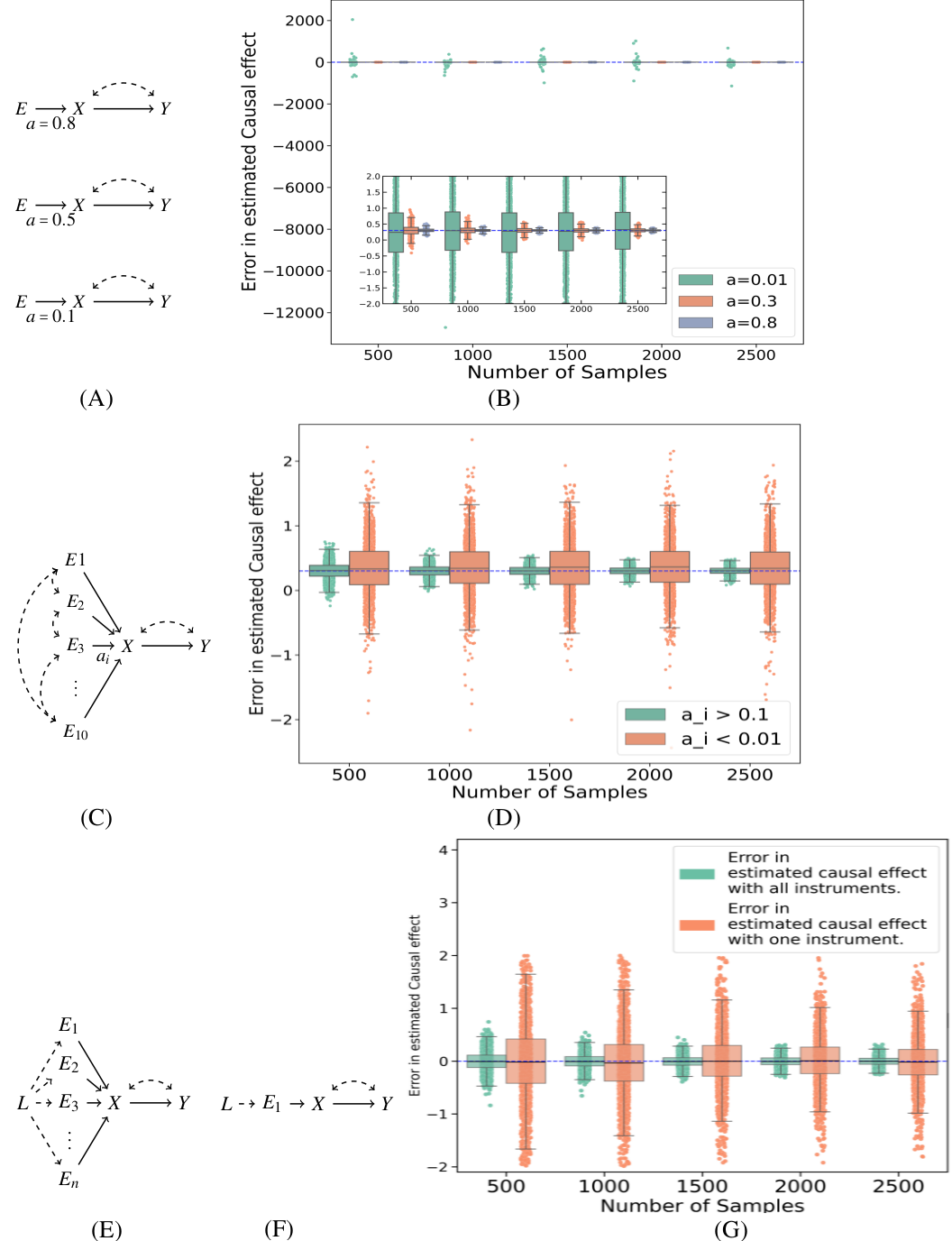

Supplement: S1 Fig — (A, C) Causal diagrams for the simulation of one exposure X for an outcome Y, influenced by one instrument E with variable instrument strengths a (A), or influenced by n ≥ 2 instruments E1, …, En with instrument strengths ai (C). (B, D) Distribution of estimated causal effects for X (true effect size 0.3), showing distributions across 1,000 independently simulated datasets across a range of sample sizes under different simulation scenarios with varying instrument strengths. (G) Distribution of estimated causal effects for X (true effect size 0.3) assuming the false diagram in (F) for inference when the true diagram that generated the data is in (E). (PNG) [file pgen.1011473.s002.png]

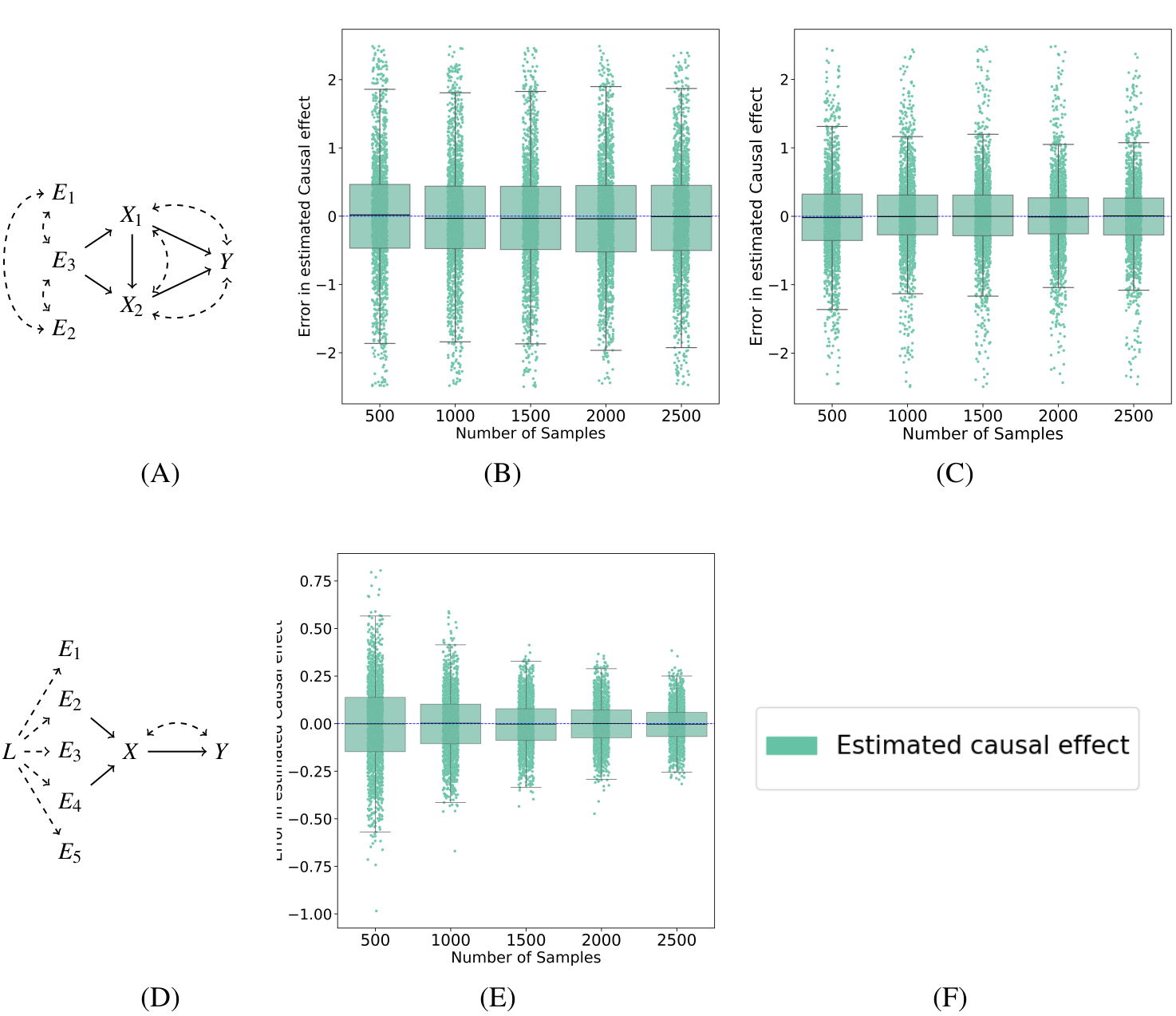

Supplement: S2 Fig — (A) Causal diagram for the simulation of two exposures X1 and X2 for an outcome Y, influenced by three instruments E1, E2, E3 where the number of causal variants is smaller than the number of cis-eGenes and other variants in the locus are merely associated by LD. (B, C) Distribution of estimated causal effects for X1 (B, true effect size 0.2) and X2 (C, true effect size 0.6) in the graph from (A). (C) Causal diagram for the simulation of one exposure X for an outcome Y, with n ≥ 2 shared instruments E1, …, En where the number of causal variants is greater than the number of cis-eGenes. (E) Distribution of estimated causal effects for X (true effect size 0.3) from the graph in (D). Panels B, C, and E show distributions across 1,000 independently simulated datasets across a range of sample sizes. (PNG) [file pgen.1011473.s003.png]

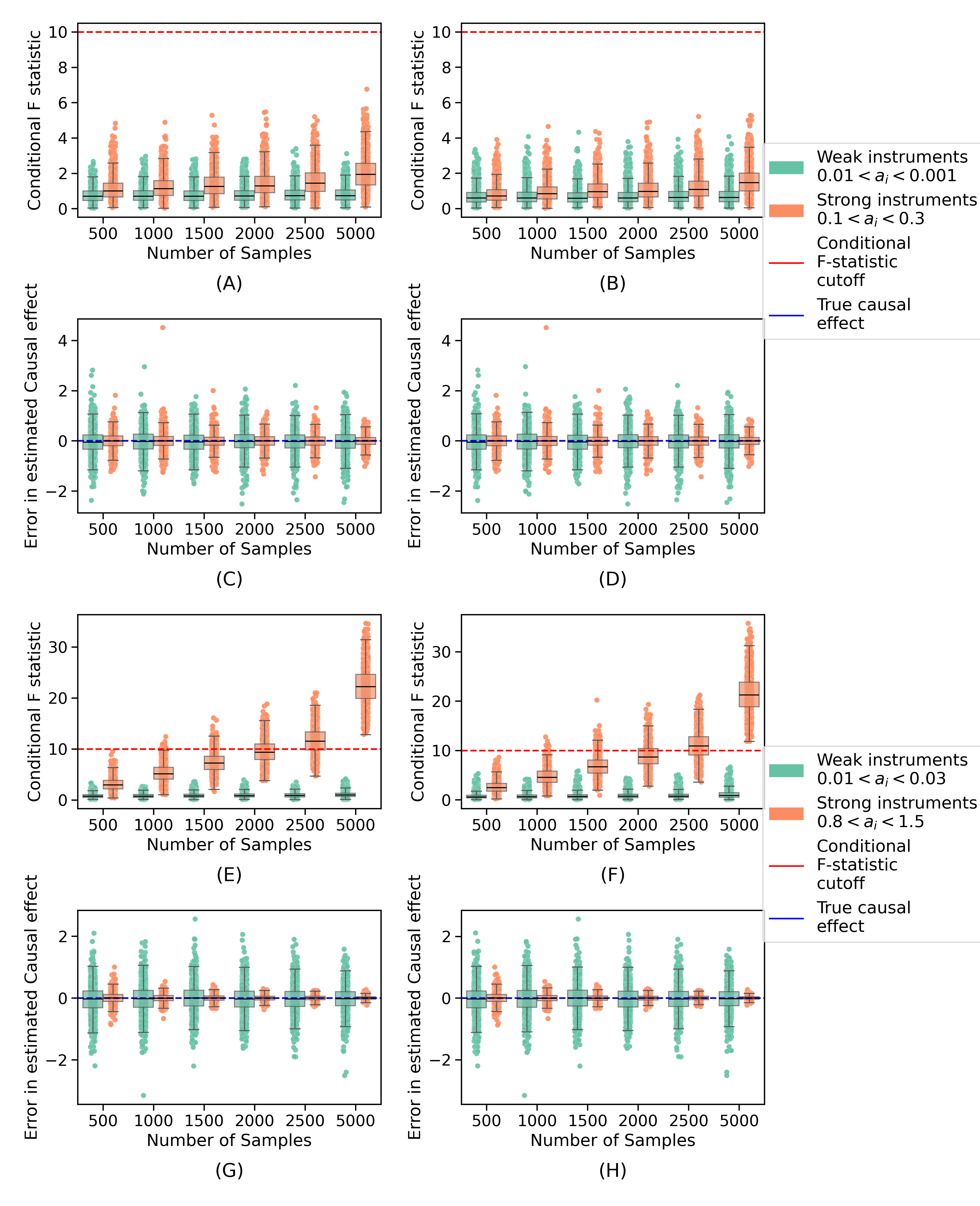

Supplement: S3 Fig — The distribution of the conditional F-statistic and causal effect estimates are shown using two effect size ranges for weak and strong instruments: 0.001 − 0.01 (weak) and 0.1 − 0.3 (strong) in subplots (A, B, C, D), and 0.001 − 0.03 (weak) and 0.8 − 1.5 (strong) in subplots (E, F, G, H). The results are based on 2,000 simulations of over-determined systems for ADAMTS7 (true effect size 0.15) where subplots (A, E) show the distribution of the conditional F-statistic using the Mendelian Randomization package and subplots (B, F) use the MVMR package. Subplots (C, G) display the distribution of causal effect estimates for the GMM estimator, while subplots (D, H) show the the distribution of the causal effect estimates for the mvivw estimator (using the Mendelian Randomization package). (PNG) [file pgen.1011473.s004.png]

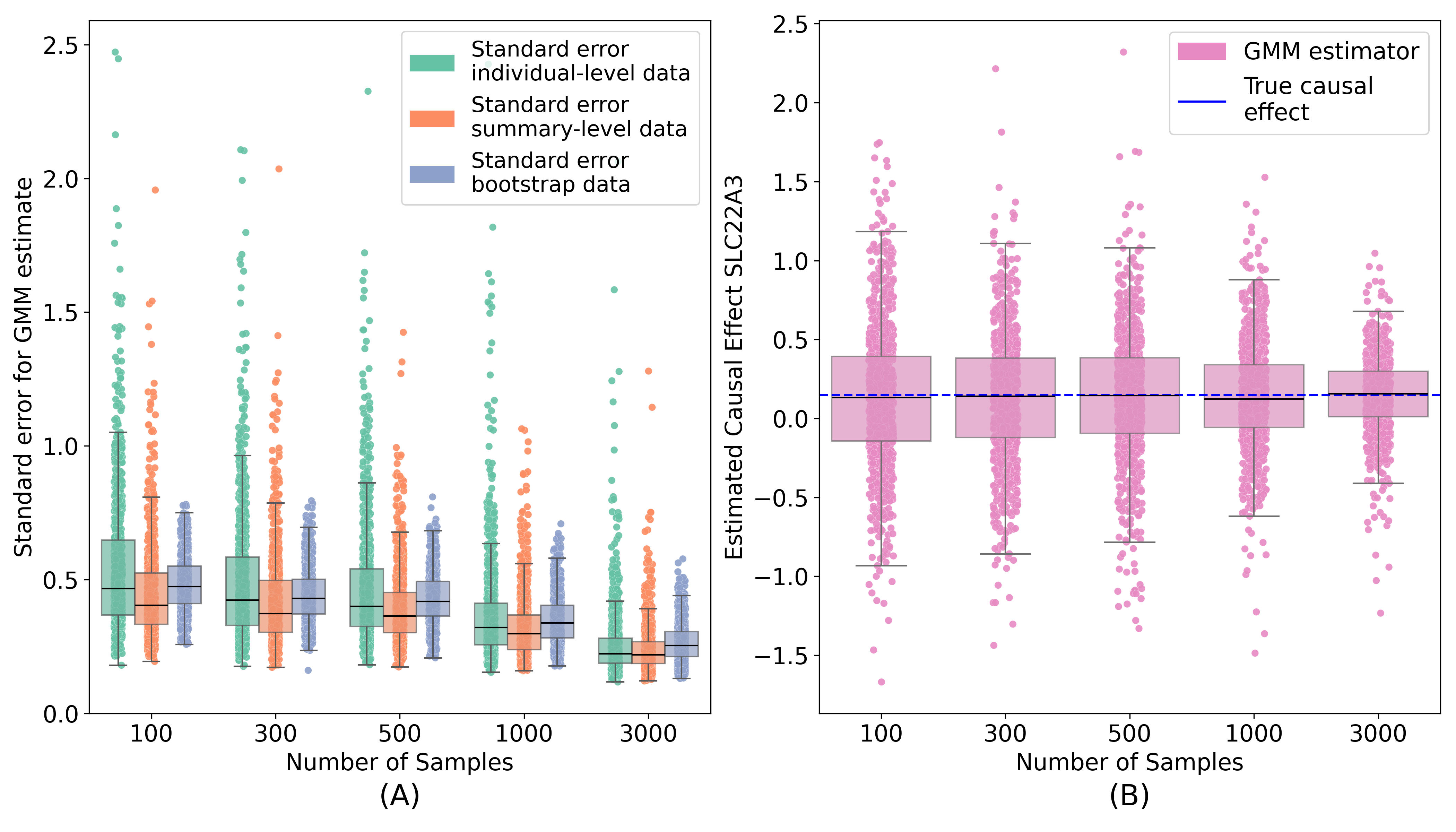

Supplement: S4 Fig — (A) Distribution of estimated standard errors for SLC22A3, using the individual level data and exact form compared to using approximate form with summary level data. (B) Distribution of estimated causal effects for SLC22A3 (true effect size 0.15) for the estimator GMM, showing distributions across 2,000 independently simulated datasets across a range of sample sizes using discrete instruments with randomly generated covariances with real LD values from the SLC22A3-LPA-PLG locus. (PNG) [file pgen.1011473.s005.png]

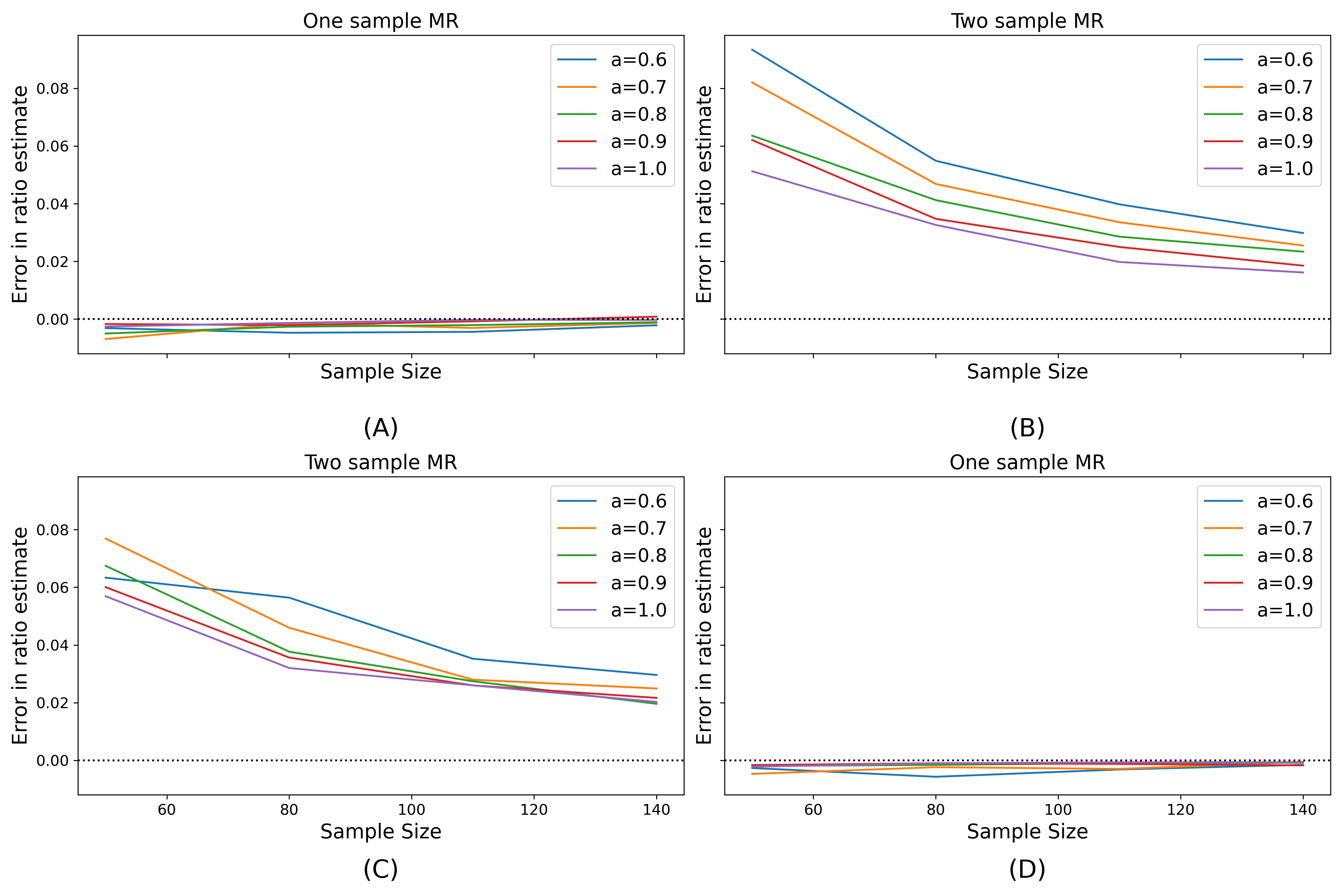

Supplement: S5 Fig — Difference (c^-c) between estimated and true causal effect of X (true effect size c = 0.8) on Y across 20,000 independently simulated datasets for a range of sample sizes comparing bias of two sample MR vs one sample MR, in a simple ratio estimate with one instrument E. (A) Cov(E, X) and Cov(E, Y) estimated from the same sample, Sample 1. (B) Cov(E, X) estimated from sample, Sample 1 and Cov(E, Y) estimated from sample, Sample 2. (C) Cov(E, X) estimated from sample, Sample 2 and Cov(E, Y) estimated from sample, Sample 1. (D) Cov(E, X) and Cov(E, Y) estimated from the same sample, Sample 2. For simplicity, sizes of Sample 1 and Sample 2 were kept the same. (PNG) [file pgen.1011473.s006.png]

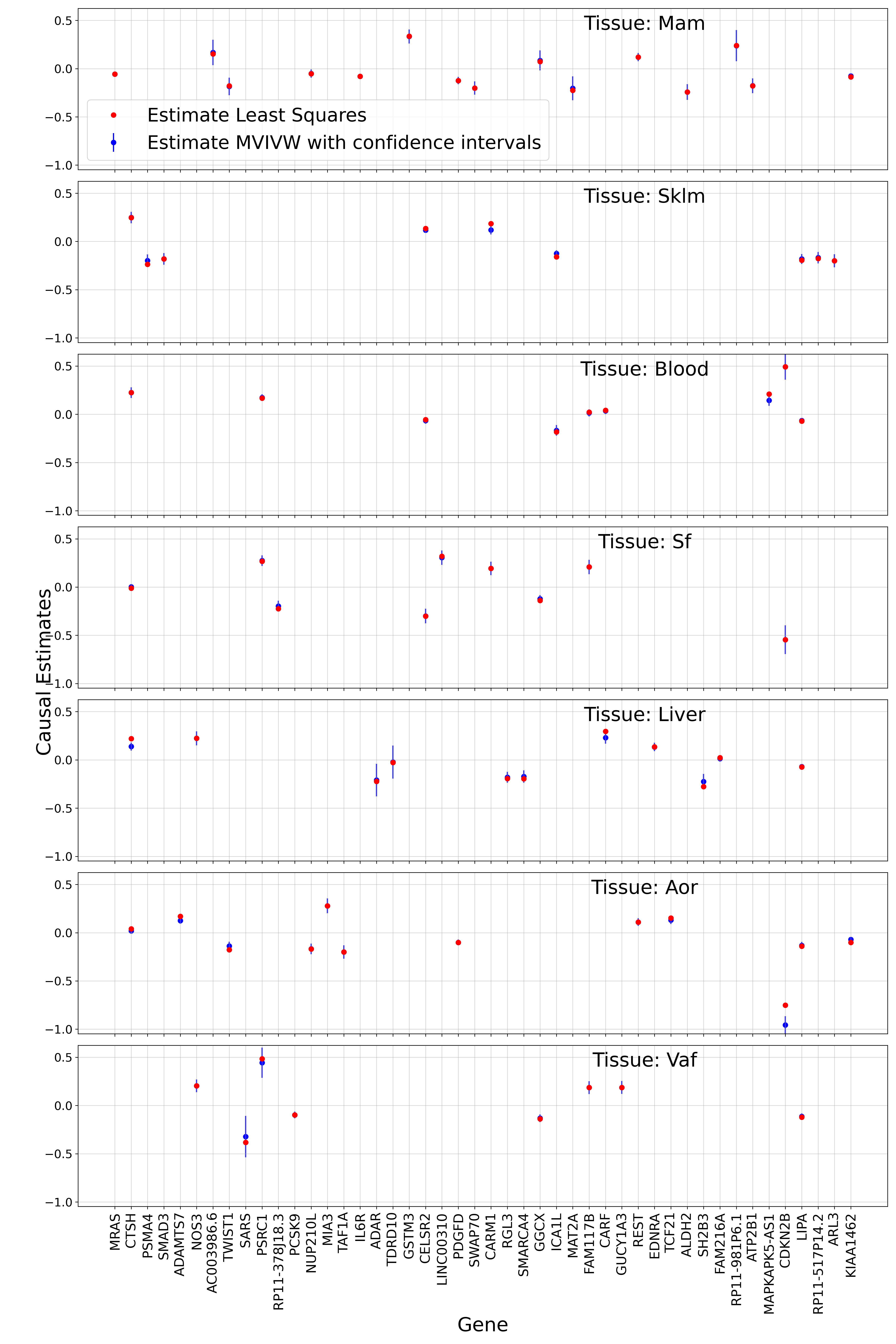

Supplement: S6 Fig — Tissue-wise causal estimates from the Least squares estimator (red) and MVIVW estimator from the Mendelian Randomization R package, with standard errors (blue). (PNG) [file pgen.1011473.s007.png]

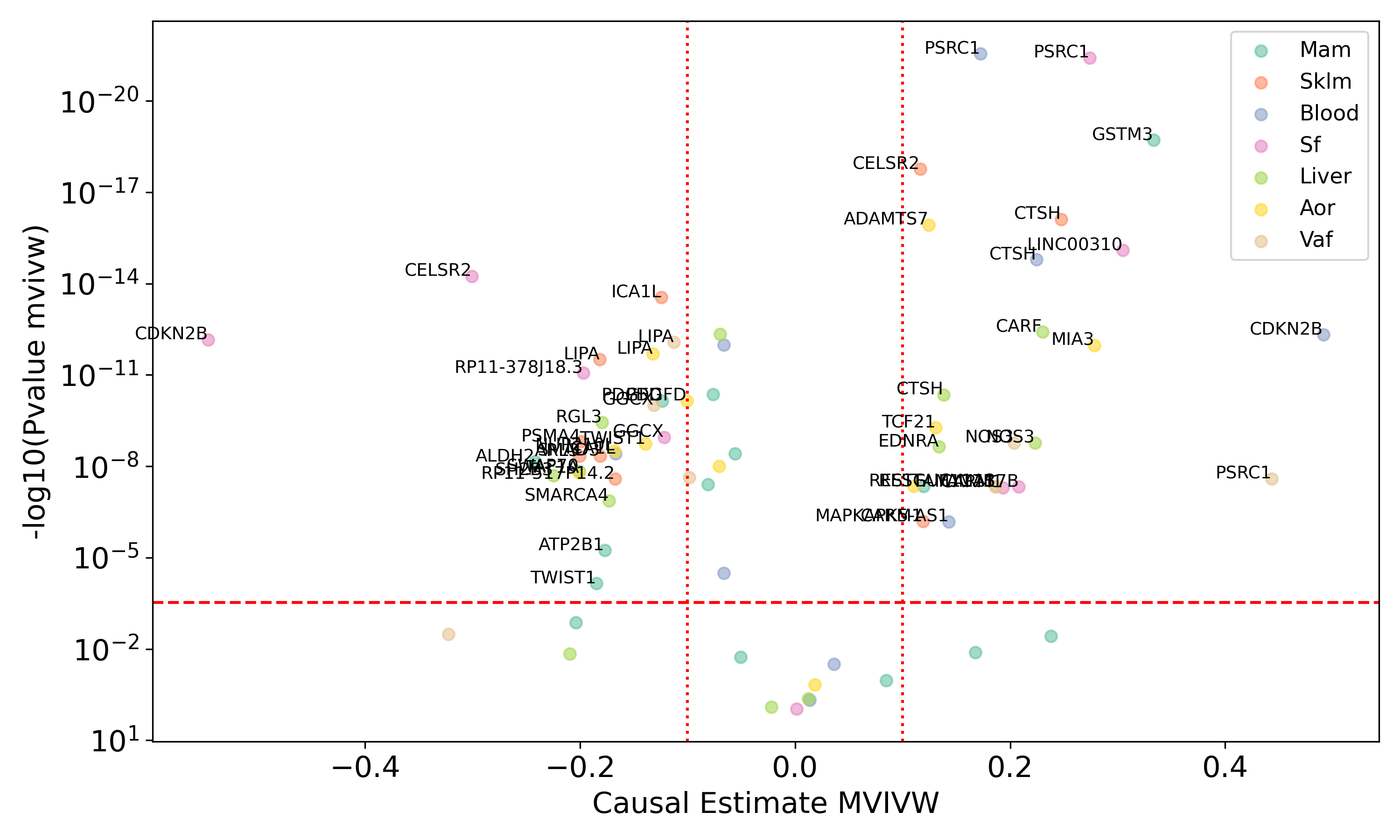

Supplement: S7 Fig — Tissue-wise causal estimates from the MVIVW estimator from the Mendelian Randomization R package, with their corresponding −log10(p − value). One outlier (CDKN2B) with estimated effect size -1.0 and p-value <10−80 not shown. (PNG) [file pgen.1011473.s008.png]

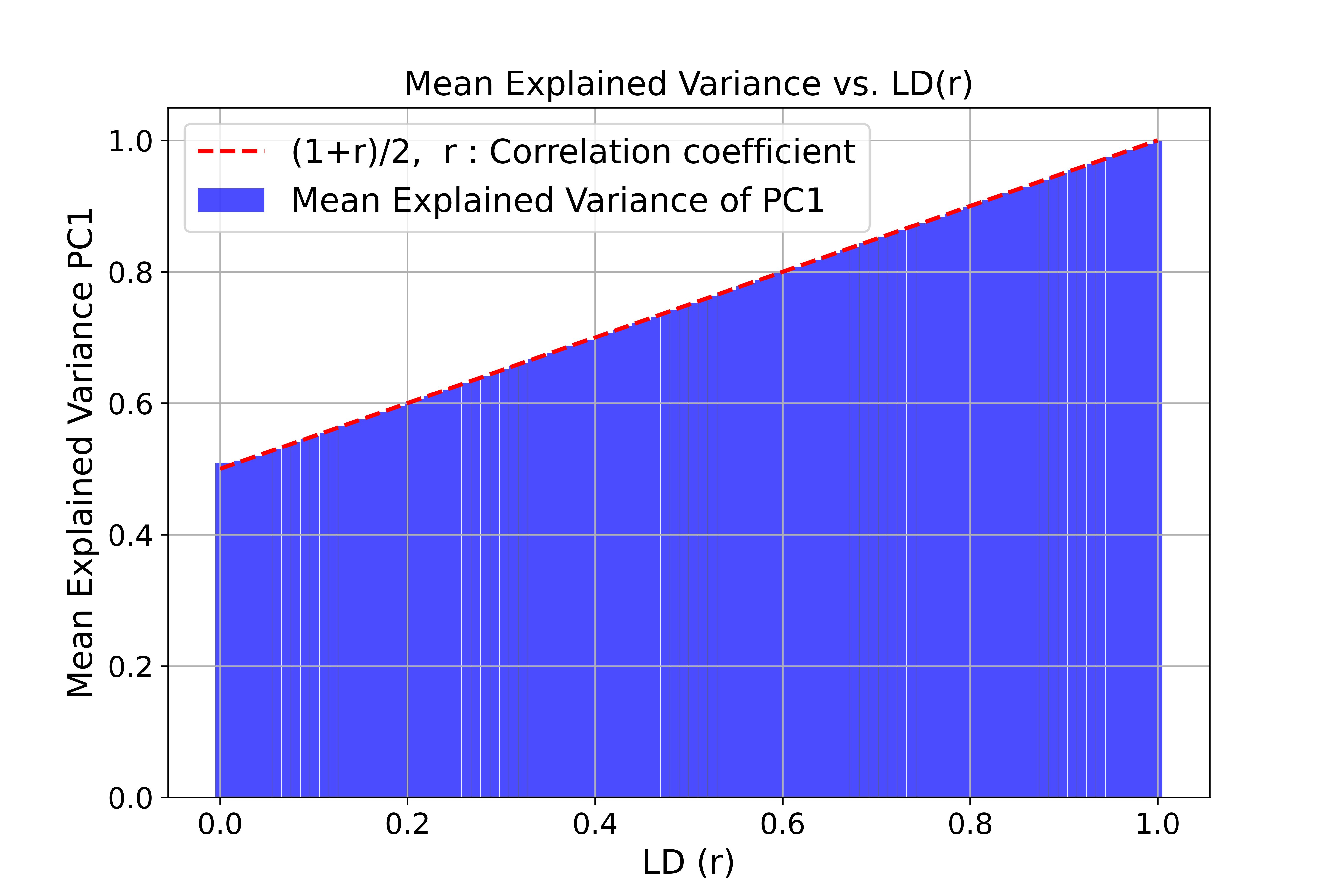

Supplement: S8 Fig — The red dashed line represents the theoretical expectation for the explained variance, calculated as 1+r2, which derives from the eigenvalues of the covariance matrix for two standardized variables with correlation r. The blue bars show the empirical mean explained variance of PC1 (Principal Component 1) obtained from simulations with a sample size of 2000 and 2000 repetitions. (PNG) [file pgen.1011473.s009.png]

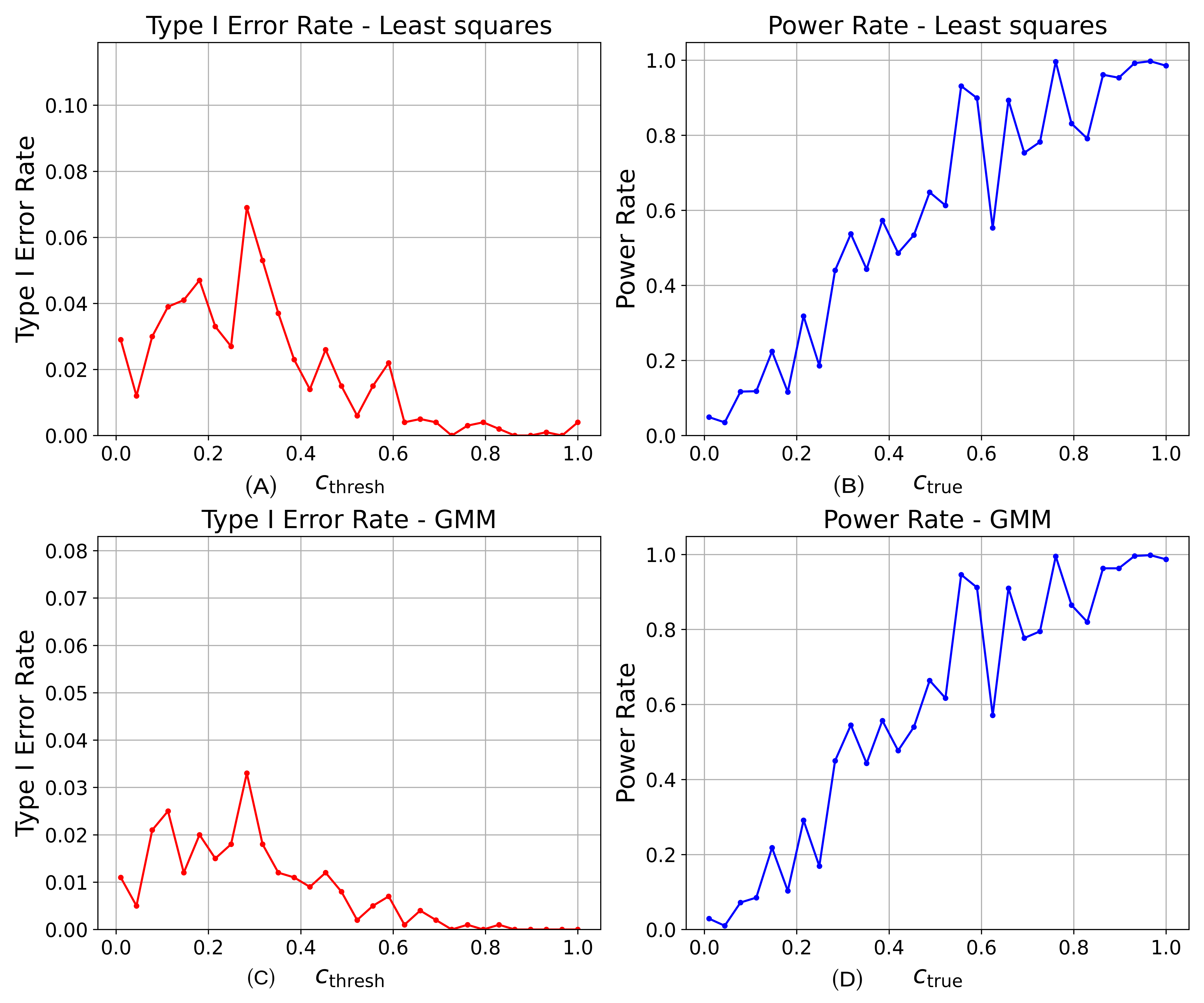

Supplement: S9 Fig — We estimate here the Type 1 error rate for the estimators Least Squares (A) and GMM (C) and Power rate for the estimators Least Squares (B) and GMM (D) from 2,000 independently simulated datasets for a fixed sample size of 2000, using discrete instruments with randomly generated covariances with real LD values from the locus on Chromosome 15:79124475 shared by genes ADAMTS7 and CTSH in the MAM tissue. (PNG) [file pgen.1011473.s010.png]
