# Supplementary material for: Prediction of causal genes at GWAS loci with pleiotropic gene regulatory effects using sets of correlated instrumental variables
Source: PLoS Genet. 2024 Nov 11;20(11):e1011473. doi: 10.1371/journal.pgen.1011473 (PMC11581411; doi:10.1371/journal.pgen.1011473)
Supplement: Attachment — Submitted filename: reviewer_response.pdf [file pgen.1011473.s011.pdf]

Response to the Editor and Reviewer comments

Editor

The reviewers appreciated the attention to an important problem, but raised substantial concerns about the current manuscript. Specific concerns revolved around the model's inability to generate a p-value, the use of an effect size cutoff of 0.1 to declare significance, and a lack of demonstration regarding its effectiveness in controlling for horizontal pleiotropic effects.

We would like to thank the editor and reviewers for their thorough assessment of our manuscript and the many helpful and constructive comments. Below we provide point-by-point responses to all comments (the original comments are copied in *blue italic*). To summarize briefly the points raised above:

- We computed standard errors of our causal estimates in the previous manuscript version but did not emphasize them or report p-values, because they are based on a one-sample estimate of the standard error, which likely underestimates the variability in the two-sample setting. To the best of our knowledge, the only standard error estimator that takes into account variablity due to the different sample sizes in the two-sample setting[1] requires individual-level data and can thus not be computed in the two-sample summary-data setting. We have now confirmed using simulations that our (one-sample) summary-based and individual-level based standard errors are comparable (Figure S4) and that our standard errors match those from the "mr_mvivw" method of the "MendelianRandomization" package (Figure S6). We have emphasized the caveat about the standard errors (and the p-values resulting from them) coming from a one-sample approximation in both the Methods and Results.
- Significance is now declared based on an absolute causal effect estimate $|\hat{c}| > 0.1$ and a p-value $p < 3 \cdot 10^{-4}$, corresponding to a conservative Bonferroni correction. We note that the additional p-value threshold removed only five gene-tissue combinations with $|\hat{c}| > 0.1$.
- We did in fact simulate the effect of horizontal pleiotropy in our previous manuscript version, albeit without clearly describing it as such. We have now expanded on these simulations, showing the bias of the estimated causal effects with increasing levels of horizontal pleiotropy. We have also performed standard tests for horizonal pleiotropy in the real data analysis from the STARNET study.

Reviewer 1

The authors present a manuscript on cis MVMR, exploring the different options through simulations and application to real data summary statistics. The methods are well-established, nothing new is proposed, but the existing ones are compared through relatively simple simulation settings. Finally, they explore transcript to CAD causal effects in various tissues in an MVMR setting and compare it to univariable MR results. The results are plausible, the methods are sound, and the conclusions are reasonably insightful. While it is not a groundbreaking paper, it represents solid work. Below I provide some comments that could help to improve the manuscript.

We thank the reviewer for their positive assessment of our manuscript and the thoughtful comments, which indeed helped to greatly improve the manuscript.

There are some key points that would be great to address, which would bring in important novel aspects and increase the impact of the manuscript:

[1] See Pacini, D., & Windmeijer, F. (2016). *Robust inference for the Two-Sample 2SLS estimator.* Economics Letters, 146, 50-54.

1. *the question of the accuracy of the LD – it is known that if we know the LD of the GWAS samples perfectly, we can include more SNPs and no need to prune aggressively, however, as the reference LD can deviate from the true LD in the GWAS (ref LD can be simulated from a Wishart distribution with the true LD), the discrepancy between the reference and target LD will cause instability in the estimation and substantial inaccuracy. This should be explored. This creates a bias-variance tradeoff: the more we prune, the lower the bias is, but the higher the variance is. However, if we prune little, the variance increases at the cost of increased bias due to LD mismatch.*

   We followed the reviewer's advice and generated new simulations were data are generated by an LD matrix that deviated from the "reference" LD matrix used in the causal effect estimation. As suggested, we sampled the study LD matrix from a Wishart distribution centred around the reference LD matrix. We simulated both one-sample settings (where eQTL and GWAS data are generated from the same sampled LD matrix) and two-sample settings (where eQTL and GWAS data are generated from different LD matrices sampled from different Wishart distributions with different variances around the reference LD matrix, to account for differences in sampling error due to sample size differences).

   We note that in the one-sample setting, the accuracy of the LD matrix only affects the generalized method of moments (GMM) estimator. The least-squares (LS) estimator only uses the SNP-gene and SNP-outcome covariance matrices (that is, summary statistics) without needing a reference LD matrix.

   The results of the new simulations are presented in Figure 3.

2. *No pleiotropy has been simulated for the instruments (could respect the INSiDE assumption). This would lead to overdispersion of the estimates, which could be explored.*

   We did in fact simulate the effect of horizontal pleiotropy, albeit without clearly describing it as such, when we simulated a scenario where the instruments are shared between two causal genes, but the estimation is done under the assumption that data for only one of them is available using univariate MR, that is, we assumed horizontal pleiotropy through an unknown/unmeasured second gene in the same locus. We have expanded on these simulations by letting the misspecified estimation model be a multivariate model, that is, by generating data using three causal genes and performing MVMR using two of them, again assuming horizontal pleiotropy through one hidden/unmeasured gene in the same locus. These new simulations clearly show how bias in the causal estimates increases with increasing levels of horizontal pleiotropy (that is, by increasing the causal effect of the "hidden" gene on the outcome).

   The results of the new simulations are presented in Figure 2.

3. *A key difficulty in cis MVMR is the estimation of the estimator variance. The authors seem to completely ignore it.*

   We computed standard errors of our causal estimates in the previous manuscript version but did not emphasize them or report p-values, because they are based on a one-sample estimate of the standard error, which likely underestimates the variability in the two-sample setting. Standard errors were computed similarly to McDaid *et al.* (2017) and p-values are now obtained from an asymptotic normal distribution for the causal estimates. We verified that the standard errors in the summary data setting (where an approximation is needed for the residual variance of the outcome) and the exact individual-level setting are comparable (new Figure S4). We also verified that our standard errors were comparable to standard errors computed by the "mr_mvivw" method of the "MendelianRandomization" package (new Figure S5). Theoretical details of the variance and p-value calculation are included in the Supplementary Information.

These theoretical calculations are valid in the one-sample setting and we acknowledge they may be over-optimistic in the two-sample setting where additional sampling error and potential bias are introduced by estimating instrument-exposure covariances from a (usually smaller) eQTL population. To the best of our knowledge, the only variance estimator for two-stage least squares causal effect estimates that accurately takes into account the different sample sizes in a two-sample setting is the one derived by Pacini and Windmeijer (2016) (see footnote 1). However their variance estimator requires individual-level data in each of the two-sample studies and is thus not applicable to the two-sample summary-data setting.

In summary, we have provided the best possible standard error estimates, but agree with the reviewer that standard error estimation in the two-sample summary-data setting remains a key difficulty in MVMR in general. We have emphasized the caveat about the standard errors (and the p-values resulting from them) coming from a one-sample approximation in both the Methods and Results.

4. *Weak instrument bias in MVMR setting can be an important issue, I do not see a detailed discussion of the compromise between the conditional F-statistic and false discoveries. Fig Supp 1b shows no attenuation of causal effect estimates (and it is looking at univariable MR only?), which is surprising. This probably means that their simulation settings are not difficult enough (and would need to be tested with multiple exposures).*

   We did compare weak and strong instruments in the MVMR setting in our previous manuscript version (Figure 2D,E) and have now expanded on this analysis. As suggested by the reviewer we have used conditional F-statistics to characterize and test for weak instruments in our simulations (Figure S3) and have reported conditional F-statistics in the supplementary tables containing the results of our analysis of the STARNET data.

5. *Methods perform very similarly (not surprising since they are somewhat equivalent), thus I'm not sure how important is to compare them against each other in the main text – I'd move cross-method comparisons [Fig 3] to the supplement.*

   We have removed these comparisons and replaced them by a new comparison showing the behavior of the methods in the presence of LD matrix bias (cf. comment 1 above).

6. *There is a growing literature on cis-MR methods, at least the latter one should be applied.*

   We applied the cis-MR / MVMR PCA method (function mr_mvpcgmm from the Mendelian-Randomization package) to our simulated data and found that it typically reported insufficient degrees of freedom even at modest degrees of LD between the instruments. To analyze this phenomenon more systematically, we focused on the simplest scenario with two instruments and two exposures. We found that at an instrument of correlation value of 0.5 or more, the PCA method reported too few (that is, only one) effective degrees of freedom for causal effect estimation. In contrast, using the LS or GMM estimators resulted in unbiased estimates with only modest increase in variance upto a correlation value of 0.9.

   These new results are presented in Figure 2.

7. *The current simulations include a very small number of SNPs only (max 4), this should be scaled up to 15-20 to reflect more real life examples.*

   We have now scaled all our simulations to 7-8 SNPs, since in our real-world data from the STARNET study we rarely found loci with more than this many SNPs after pruning with LD threshold 0.85-0.99. There was one locus 15:79124475 that had 25 SNPs at LD threshold 0.95, but generating simulated data using the real LD matrix at this locus invariably resulted in instruments with a singular sample covariance matrix. We therefore considered the simulations with 7-8 SNPs sufficiently representative of real-life examples.

8. *Lot could be cut/moved to the supplement in the sections "Causal effect identification in MVMR with correlated instruments" and "The generalized method of moments for causal effect estimation in MVMR with correlated instruments"*

   We appreciate the suggestion, but have kept the sections as they were, as there is no length restriction from the journal and the other reviewers did not make comments on this. We would however be happy to reconsider if the consensus is that these sections should be reduced.

9. *I'm not sure about the sample sizes used in the simulations. I see 500-2500, but typically the outcome GWAS would be much larger in terms of sample size. Were both sample sizes (eQTL snd GWAS) varied?*

   In our previous simulations the same sample size was used to simulate eQTL and GWAS data. We have now performed simulations where the eQTL sample size varied between 300-4000 and the GWAS sample size varied between 10000-140000, in line with current real-world sample sizes. These new simulations have the additional benefit of mimicking a two-sample scenario, since the exposure values in the eQTL data are now sampled independently of the (hidden) exposure values used to generate the outcome values in the GWAS data.

   The results of the new simulations are presented in Figure 3.

10. *In the real data application, some gold standard could be derived from orthogonal data to test whether MVMR pinpoints relevant genes? (E.g. drug target genes are more likely to be true hits or OMIM genes for monogenic forms of heart disease or CAD-related rarer diseases.)*

    We appreciate the suggestion. One reason why we restricted our analysis of the real data to genome-wide significant CAD loci is that the candidate genes in these loci have been previously analyzed using exactly the kind of orthogonal data suggested by the reviewer [see e.g. Braenne *et al.*, ATVB 35:2207 (2015); Hao *et al.*, Circ Genom Precis Med 15:e003365 (2022)]. Thus, the arguments presented in our manuscript about cases where MVMR pinpoints the "correct" gene are indeed based on such analyses, albeit indirectly through these cited studies.

11. *Minor comments*

    (a) *The font is too small in the legends (e.g. Fig2)*

    We assume font sizes etc. will be typeset by the published and have therefore kept the default settings of the template.

    (b) *The locuszoom plots in Fig 5 are not really informative and hard to read. I'd remove them.*

    The plots are meant to illustrate the complexity of regulatory pleiotropy within a genomic locus, with different linked SNPs being associated with different combinations of genes within the same locus. We agree they are a bit hard to read, but have not found a better way of representing this information.

    (c) *McDaid and Porcu et al do not propose two-stage least squares estimators, but two-sample MR estimators.*

    To be precise, McDaid *et al.* show that the two-stage least squares estimator can be computed using summary statistics (cf. their Methods section, *"Using two-stage least squares, ..."*), which indeed results in a two-sample estimator. We clarified this in the Introduction.

Reviewer 2

The authors introduce a MVMR method with correlated instrumental variables to identify the candidate causal genes at GWAS loci. Overall, the manuscript is well organized, especially with the rigorously theoretical justification. The simulations and real data analyses show good performance. However, I have some comments that the authors should be addressed, with details listed below.

We thank the reviewer for their positive assessment of our manuscript and the thoughtful comments, which helped to greatly improve the manuscript.

1. *The authors should clarify the conceptual item "regulatory pleiotropy", and elucidate how it differs from horizontal pleiotropy in traditional MR. Although utilizing molecular traits as exposures in MR can mitigate the risk of horizontal pleiotropy, concerns about pleiotropic bias remain. It would arise if variants were involved in different biological pathways relevant to the outcome, resulting in heterogeneous causal effect estimates. Intuitively, for the correlated IVs, only a small subset of them to be causal, while others will elevate the risk of horizontal pleiotropy. In addition, I suggested the authors to explore the impact of horizontal pleiotropy in the simulations.*

   We borrowed the term "regulatory pleiotropy" from the GTEx Consortium 2020 paper [GTEx Consortium, Science 369:1318 (2020)] who defined *cis*-eVariants with regulatory pleiotropy as those associated with more than one gene in the same locus. Thus defined, regulatory pleiotropy implies horizontal pleiotropy in a univariate MR where only one of the genes is considered as an exposure. We have now clarified this distinction in the Introduction.

   Taking regulatory pleiotropy into account as we did in our manuscript indeed does not exclude that horizontal pleiotropy is still present, and we noted an important case in our manuscript, where a variant may affect gene expression in cell types or tissues for which no data is available. We have further emphasized this point in the Discussion.

   We did in fact simulate the effect of horizontal pleiotropy, albeit without clearly describing it as such. Previously we simulated a scenario where the instruments are shared between two causal genes, but the estimation is done under the assumption that data for only one of them is available using univariate MR, that is, we assumed horizontal pleiotropy through an unknown/unmeasured second gene in the same locus. We have expanded on these simulations by letting the misspecified estimation model be a multivariate model, that is, by generating data using *SLC22A3-LPA-PLG* causal genes and performing MVMR using two of them *LPA-PLG*, assuming horizontal pleiotropy through one hidden/unmeasured gene *SLC22A3* in the same locus. In these new analyses, the magnitude of the causal effect of *SLC22A3* on the outcome was varied to test how bias in the causal effect estimates of *LPA-PLG* depends on the degree of horizontal pleiotropy.

   The results of the new simulations are presented in Figure 2.

2. *Is it possible to obtain p-values using the derived least-square estimator or the GMM estimator, given that the p-value can also be obtained in MVMR and TWMR. It is quite uncommon to declare the significance only based on the absolute causal effect size greater than 0.1, rather than the p-value.*

   We computed standard errors of our causal estimates in the previous manuscript version but did not emphasize them or report p-values, because they are based on a one-sample estimate of the standard error, which likely underestimates the variability in the two-sample setting. To the best of our knowledge, the only standard error estimator that takes into account variablity due to the different sample sizes in the two-sample setting (see footnote1) requires individual-level data and can thus not be computed in the two-sample summary-data setting. We have now confirmed using simulations that our (one-sample) summary-based

and individual-level based standard errors are comparable (Figure S4) and that our standard errors match those from the "mr_mvivw" method of the "MendelianRandomization" package (Figure S6). We have emphasized the caveat about the standard errors (and the p-values resulting from them) coming from a one-sample approximation in both the Methods and Results.

Significance is now declared based on an absolute causal effect estimate $|\hat{c}| > 0.1$ and a p-value $p < 3 \cdot 10^{-4}$, corresponding to a conservative Bonferroni correction. We note that the additional p-value threshold removed only five gene-tissue combinations with $|\hat{c}| > 0.1$.

3. *For the simulation with randomly generated instrument correlations, based on the Fig. 1A, what is the instrument strength a? Is it the effect size of the IV on the exposure? In addition, it seems to be confusing about the determinant of the instrument effect size matrix A. Why is the determinant of A directly associated with the instrument strength? For example, in a scenario with two IVs, if the effect size of the first IV on the exposures is 0.5 and 0.4, and for the second IV it is 0.4 and 0.5, the determinant of A is calculated as 0.5 * 0.5 - 0.4 * 0.4 = 0.09, indicating weak instruments. However, each instrument's effect size is greater than 0.4, which can be view as the strong instrument.*

   We thank the reviewer for this comment. We have now changed our simulations and sample instrument strengths randomly. For strong instruments, we take effect sizes greater than 0.5 and for weak instruments we take effect sizes smaller than 0.03. We further confirmed that the sampled instruments are indeed weak or strong using conditional F- statistic (Figure S3).

   The results of the new simulations are presented in Figure 2.

4. *For the simulation with instrument correlations derived from real LD matrices, the assumption that all eQTLs are causal for all genes may be unrealistic. The causal effect of eQTLs are more likely to be sparse. In addition, the two nearby genes are practically impossible to be the causal gene simultaneously. It is important to assess the performance of the proposed method when the genetic architecture is sparse.*

   We thank the reviewer for this important comment. All our simulations with overdetermined systems now assume that only a randomly selected subset of at least K eQTLs ($K \geq$ number of exposures) were causal and all others were associated with the exposures only through LD.

5. *In the real data application, the eQTL summary statistics is from CAD patients. It raises concerns about whether this data is suitable for evaluating the gene expression effect on CAD. The interpretation may be limited in the CAD patients rather than reflecting the general causal effect of a specific gene on CAD.*

   We appreciate the concern of the reviewer. While predicting CAD risk using expression data from CAD cases can be criticized, we have shown previously that more gene expression traits are associated with CAD risk loci in STARNET cases than in comparable disease-unspecific samples (e.g., GTEx) [Franzén *et al.*, Science 353:827 (2016)], that eQTLs inferred from CAD cases explain a large proportion of heritability of CAD risk [Zeng *et al.*, JACC 73:2946 (2019); Koplev *et al.* Nature CVR 1:85 (2022)], and that variation in eQTL-associated genes and gene networks correlates with the extent of atherosclerosis in CAD cases [Talukdar *et al.*, Cell Syst 2:196 (2016); Koplev *et al.* Nature CVR 1:85 (2022)], suggesting that variation in gene expression and disease severity in CAD cases can indeed reflect underlying causal effects on CAD risk. This was further supported by the finding in this manuscript that causal effects estimated from STARNET and GTEx samples for the same genes and tissues correlated well.

We have added the justification above to the Discussion.

In addition, why the authors focus on the genome-wide significant GWAS loci to perform MVMR analysis and use SNPs with GWAS $p-value \leq 5e-08$ as IVs? In the traditional MR analysis, researchers often exclude SNPs significantly associated with the outcome to avoid violating the exclusion restriction assumption.

We focused our analysis on genome-wide siginificant GWAS loci because the use of eQTL information to identify candidate genes is an important part of modern GWAS protocols [see e.g. Uffelmann *et al.*, Nat Rev Methods Primers 1, 59 (2021)] and because candidate genes at many GWAS loci have been analyzed using orthogonal data, providing some form of validation for our approach. Needless to say, our approach also could be applied to loci passing less stringent thresholds or be integrated in TWAS fine-mapping methods (cf. comment below). We have added this explanation to the Discussion.

To mitigate issues related to exclusion restriction (horizontal pleiotropy) in the STARNET data analysis, we have used the *Cochran's Q-statistic* using the *Mendelian Randomization* package and reported it in the supplementary tables with the results of our analysis of the STARNET data.

Another consideration is that gene expression is estimated to mediate about 10–20 percent of trait heritability. It may exist some SNPs through alternative pathways affect the CAD in one specific loci.

The presence of horizontal pleiotropy is always a possibility in any MR-based study, and we emphasized an important caveat in our manuscript, where a variant may affect gene expression in cell types or tissues for which no data are available. We have reaffirmed this point in the Discussion.

6. *Many TWAS fine-mapping method have proposed to model all pleiotropic genes in one genomic region, like FOCUS (Mancuso, N., Nat Genet, 2019), cTWAS (Zhao, s., Nat Genet, 2024), GIFT (Liu, L., Nat Genet, 2024). I wonder whether the theoretical framework proposed in this manuscript could be extended to these TWAS fine-mapping methods. The authors should, at least, discuss this.*

   We thank the reviewer for the suggestion. Integrating our approach with TWAS fine-mapping methods would indeed be a logical and exciting next research step. These fine-mapping methods typically use sparsity-inducing constraints to predict outcome traits from a minimal set of most informative genes, based on the hypothesis that the selected genes will most likely be the causal genes, but without explicit causal DAG modelling. Using results from a causal model such as considered here, either directly in an outcome prediction model, or indirectly as part of the sparsity constraints, could increase the probability that the minimal predictive gene set indeed consists of truly causal genes.

   We have added this point to the Discussion.

7. *Please clarify the impact of LD matrix calculation. Specifically, is the LD matrix derived from in-sample LD or an external reference panel? Is MVMR with correlated IVs sensitive to the difference between the LD reference panel and the GWAS data? What is the performance if we use the external reference panel, such as 1000 Genomic Project?*

   In our analysis of the STARNET data, we presented causal effect estimates from the least-squares (LS) method in the main text. The LS method only needs eQTL and GWAS summary statistics, and no in-sample or external LD matrix is required.

   We also computed causal effect estimates in STARNET using the generalized method of moments (GMM). In this case we used LD matrices from the 1000 Genomes project. Both

methods (LS and GMM) resulted in comparable causal effect estimates (see the Supplemantary Tables deposited on Dataverse.no).

Prompted by this comment and related comments by reviewer 1, we revisited some of our simulations, since our previous simulations were using the same "in-sample" LD matrix for both eQTL and GWAS data. We now generated new simulations were data are generated by an LD matrix that deviated from the "reference" LD matrix used in the causal effect estimation. As suggested by reviewer 1, we sampled the study LD matrix from a Wishart distribution centred around the reference LD matrix. We simulated both one-sample settings (where eQTL and GWAS data are generated from the same sampled LD matrix) and two-sample settings (where eQTL and GWAS data are generated from different LD matrices sampled from different Wishart distributions with different variances around the reference LD matrix, to account for differences in sampling error due to sample size differences).

The results of the new simulations are presented in Figure 3.

8. *Minor Comments*

   (a) *Please check the citation of figures, as there are multiple errors noted, particularly in Fig. 5B.*

   We have thoroughly checked the manuscript text and figure captions and hope to have corrected all errors.

   (b) *The description of the datasets is unclear. For example, the authors should explicitly state the sample sizes of exposure and outcome data used in both the simulation and real data applications.*

   The sample sizes for all simulations are now mentioned throughout. We also added the sample sizes of the exposure and outcome data in the CAD application to the Methods section.

Reviewer 3

The authors investigated an interesting topic of using correlated IVs in multivariable Mendelian randomization (MVMR) analysis. They showed the identification of causal effect in MVMR with the use of correlated IVs by applying the method of path coefficients. And they proposed a GMM estimator to estimate the causal effects, which has also been shown to be equivalent to two-stage least square estimator. I have concern in novelty and soundness of the current study.

We thank the reviewer for their positive assessment of our manuscript and the thoughtful comments, which helped to greatly improve the manuscript.

1. *My major concern is that as a causal inference method, the authors did not try to do any statistical inference on the causal estimator. The authors should propose a hypothesis testing procedure.*

   We computed standard errors of our causal estimates in the previous manuscript version but did not emphasize them or report p-values, because they are based on a one-sample estimate of the standard error, which likely underestimates the variability in the two-sample setting. To the best of our knowledge, the only standard error estimator that takes into account variablity due to the different sample sizes in the two-sample setting (see footnote1) requires individual-level data and can thus not be computed in the two-sample summary-data setting. We have now confirmed using simulations that our (one-sample) summary-based and individual-level based standard errors are comparable (Figure S4) and that our standard

errors match those from the "mr_mvivw" method of the "MendelianRandomization" package (Figure S6). We have emphasized the caveat about the standard errors (and the p-values resulting from them) coming from a one-sample approximation in both the Methods and Results.

2. *Related to the previous point, the current way to determine the presence of a causal relationship is based on some heuristic thresholds, e.g. 0.1 was used in the real data application. I strongly doubted the efficacy of such a heuristic method, which is based on the fact that the authors have already had a glimpse of the data and then determined the threshold. Again, a proper statistical testing should be used instead of using arbitrary thresholds.*

   Significance is now declared based on an absolute causal effect estimate $|\hat{c}| > 0.1$ and a p-value $p < 3 \cdot 10^{-4}$, corresponding to a conservative Bonferroni correction. We note that the additional p-value threshold removed only five gene-tissue combinations with $|\hat{c}| > 0.1$.

3. *In the real data application, I don't understand why the authors selected IVs also based on the GWAS significance with the outcome CAD, while most MR studies would just select IVs simply based on their association with the exposure. Could the authors explain why? This may be problematic. First, it is very likely that pleiotropic variants would be included, if an IV is strongly associated with the outcome (perhaps even more significant than the IV-exposure association; for example, see Steiger's filtering [1]); Second, selecting IVs based on the association with the outcome can introduce winner's curse, and in the two-sample MR scenario, a upward bias in the causal estimator. This may have an impact in the current study design as the author used a threshold to determine causal gene.*

   We focused our analysis on genome-wide siginificant GWAS loci because the use of eQTL information to identify candidate genes is an important part of modern GWAS protocols [see e.g. Uffelmann *et al.*, Nat Rev Methods Primers 1, 59 (2021)] and because candidate genes at many GWAS loci have been analyzed using orthogonal data, providing some form of validation for our approach. Needless to say, our approach also could be applied to loci passing less stringent thresholds or be integrated in TWAS fine-mapping methods (cf. comment below). We have added this explanation to the Discussion.

   To mitigate issues related to exclusion restriction (horizontal pleiotropy) in the STARNET data analysis, we have used the *Cochran's Q-statistic* using the *Mendelian Randomization* package and reported it in the supplementary tables with the results of our analysis of the STARNET data.

   Finally, as indicated above, we now used a p-value threshold in addition to an estimated causal effect size threshold to determine causal genes.

4. *As the authors also point out, their proposed GMM estimator is the same as TSLS, what is the major novelty/contribution in this study? The authors should further clarify this to strengthen their work.*

   The main novelty is that we use Wright's method of path coefficients to prove mathematically that causal effect identification and estimation in MVMR can be achieved with (highly) correlated instruments and is not limited to independent or weakly dependent instrument sets. This is particularly important when MVMR is applied to determine causal genes at genomic loci with regulatory pleiotropy, where SNPs are by definition in high LD. Furthermore our study is the first to perform simulations for MVMR with correlated instruments, which unambiguously confirmed the validity and usefulness of the theoretical results even at modest sample sizes. We have reaffirmed these points in the Introdcation and Discussion.

5. *In the simulation study, the authors should consider simulate IVs from a binomial distribution to reflect the nature of genotypes in MVMR. The authors mentioned in lines 300-302 that "...is not practical in our simulation, because the variables must be correlated according to a predefined LD matrix..." The authors may want to consider estimating the LD matrix based on their simulated genotypes, or even simulating another set of individuals to be used as a reference panel. Related to this, how did the authors estimate the LD matrix in their real data application?*

   We agree with the reviewer that more realistic simulations with discrete, binomially distributed genotypes were required. However we wish to remind the reviewer that our goal is to simulate genotypes for *correlated* instruments (with a given LD matrix as input), whereas all simulations reported in the MVMR literature so far have been for *independent* SNPs. We used normally distributed instruments in our initial simulations, because it is trivial to sample from a multivariate normal with pre-specified covariance (LD) matrix. In contrast, we are not aware of a simple method to do the same for binomially (or even Bernoulli) distributed variables.

   To overcome this challenge and perform simulations with discrete genotypes, we compromised between independent and completely arbitrary LD matrices, and assumed that the correlation structure between the simulated instruments can be described by a Markov chain. In other words, we sample the first SNP's genotype from a binomial distribution with probability of success its minor allele frequence (MAF). For every other SNP, a genotype is sampled from a binomial distribution with probability of success conditional on the genotype of the previous SNP in the sequence, such that both the MAF and pairwise LD between successive SNPs match the real data from the simulated locus (see details in the manuscript and Supplementary Information). All our simulations now used this approach to generate instrument data.

   For the real data we used reference LD matrices from the 1000 Genomes project.

6. *It would be helpful if the authors can add some simple numeric examples based on a graph to illustrate their method, especially for Eqs. (1), (2).*

   As an example, we have added explicit solutions for the one and two-exposure cases to the Supplementary Information, using the method of path coefficients, verifying the instrumental set conditions, and showing how the same result is obtained by a more tedious manipulation of the underlying SEM. We hope this clarifies the equations.

7. *In Figure S2 scenario (A) only one causal instrument is available for two exposures, which is described as an 'underdetermined' model, why is the performance of method still perfect? Is this contradict to page 10 line 402-404 "However, if the number of true causal variants is less than the number of exposures... does not help"*

   Although it may not be obvious visually due to the high variance in the estimates, the performance is in fact *not* perfect. The causal estimates are biased (non-zero deviation from the true value), and the variance in the estimates is about ten-fold greater than in the exactly determined model (compare Figure 2 and Figure S2).

8. *Minor comments*

   (a) *Typo: line 354, "LD greater than 0.0"*

   We rephrased this as *"...all eQTLs within a 0.5Mb radius and with non-zero LD with the lead SNP."* In other words, for the real data analysis in the paper we primarily selected eQTLs based on a distance cutoff without requiring a minimum LD with the lead SNP. We note that our online software contains a threshold parameter that can

be easily set to a non-zero value to select eQTLs based on both distance and minimum LD with the lead SNP.

(b) *I am confused about "both p_i[V Y] and p_j[E_j V] point to V" in line 182. It would help if an example were provided.*

We have clarified this using the new examples in the Supplementary Information mentioned above.
