# Supplementary material for: Prediction of causal genes at GWAS loci with pleiotropic gene regulatory effects using sets of correlated instrumental variables
Source: PLoS Genet. 2024 Nov 11;20(11):e1011473. doi: 10.1371/journal.pgen.1011473 (PMC11581411; doi:10.1371/journal.pgen.1011473)
Supplement: Attachment — Submitted filename: reviews.pdf [file pgen.1011473.s012.pdf]

Response to the Reviewer comments

Reviewer 1

The authors made a great effort to address my comments and suggestions - I appreciate it. I have only a few remaining minor comments left to be addressed, mostly regarding Figures:

We thank the reviewer for their appreciation and constructive comments and insights throughout the review process, which have helped to greatly improve the manuscript.

1) Fig 2B: Y axis label should be replaced to "Bias of the estimated causal effect..."

In Fig. 2B, the Y-axis is labeled "Error in estimated causal effect for Gene 1." In this and all other simulations, we plot the difference between the true causal effect and the estimated causal effect. To maintain consistency, we refer to this difference as "error" rather than "bias."

2) Fig 2C: I'd remove this panel since it is common sense that the explained variance of the first PC for two variables with correlation r is (1+r)/2.

We have removed this panel and have instead briefly explained the mentioned result in the SI text and Fig S8, showing the theoretical expectation for the explained variance, which derives from the eigenvalues of the LD-matrix, against the empirical mean explained variance of principal component 1 obtained from simulations with a sample size of 2,000 and 2,000 repetitions.

3) Fig 2D: The outer plot is not needed, since it only shows the outliers, the real information is in the inner plot. Also, weak instrument bias would bias the estimates towards the null, thus I'm surprised to see no bias in the effect estimation. Maybe the weak instrument bias is too small (i.e. the settings are too easy)?

We thank the reviewer for this comment. We have firstly changed all simulations (except Fig3 (E,F)) using new effect sizes for strong and weak instruments. For over-determined cases where we have more instruments than exposures we sampled instrument effect sizes randomly between $0.001 - 0.01$ (weak instruments) and $0.1 - 0.3$ (strong instruments). We have also updated the F-statistic simulation to include the new effect sizes in Figure S3. For the exactly determined case where we have two instruments and two exposures, we sampled the matrix $A = [a_{ij}]$ of instrument effect sizes, where a_{ij} is the direct causal effect of E_i on X_j, randomly such that (a) its determinant was greater than 0.05 and there were no weak instruments (a_{ij} between $0.1 - 0.3$) (strong instruments), and (b) its determinant was less than 0.001 and effect sizes of each instrument less than 0.001 (weak instruments).

We have also changed the plot in Fig 2D using the new effect sizes for strong and weak instruments while also removing the outer plot. The weak instrument bias is now more apparent with the updated effect sizes and removal of the outer plot.

4) The fact that the error in the LD does not lead to any bias is unexpected, but I guess it stems from the fact that the exposure and outcome effect estimates were simulated from the same LD structure. This could be added as a Discussion point.

We have added a sentence to the Discussion to highlight this point.

Reviewer 2

I thank the authors for their efforts and hard work. I'm very happy with their replies to my previous comments and suggestions. However, I still have some comments that may need an additional attention.

We thank the reviewer for their appreciation and constructive comments and insights throughout the review process, which have helped to greatly improve the manuscript.

1. To my knowledge, it is possible to use the rank inversion technique, or resampling methods to obtain the standard error. I wonder if these approaches would be better than the approximation from one-sample estimation.

We appreciate the reviewer's suggestion. In addition to approximating standard errors from one-sample estimations, we have now applied bootstrapping methods to estimate the standard errors of our causal estimates. Specifically, for the regression of SNPs on genes, we generated bootstrapped effect sizes by drawing from a multivariate normal distribution centered on the observed effect sizes, using the covariance matrix derived from independent instruments. This approach assumes that genes are conditionally independent given the SNPs. Similarly, for the regression of SNPs on CAD, we used the same bootstrapping approach based on the observed effect sizes and their covariance structure from independent instruments. A detailed derivation of the distribution of effect estimates can be found in the SI Methods. The bootstrap estimates are included in Figure S4, where it can be seen that they tend to be higher than the true values, particularly at larger sample sizes.

To be of practical relevance for our method, the bootstrap method should be extended to correlated instruments, but further research is required to derive the correct distribution of the causal estimates in this case. We note that the procedure we implemented is an instance of a *parametric* bootstrap. A *non-parametric* bootstrap would not require knowledge of the distribution of causal effects, but cannot be applied in the summary-data setting because this would require individual-level data for generating bootstrap samples.

2. It would be beneficial to compare the proposed least squares and GMM estimators to other existing MVMR methods, such as MVMR-Robust, MVMR-Median, and MVMR-Lasso (Grant AJ, Stat Med, 2021), MR-BMA (Zuber V, Nat Commun, 2020), and MVMR-cML (Lin Z, Am J Hum Genet, 2023).

We thank the reviewer for the suggestion. We have now added new simulations in Fig 3 (G,H,I) where we show the comparison of least squares and GMM estimators to MVMR-Robust, MVMR-Median, and MVMR-Lasso (Grant AJ, Stat Med, 2021), and MVMR-cML (Lin Z, Am J Hum Genet, 2023). We have not compared the estimators to MR-BMA (Zuber V, Nat Commun, 2020) as we were not able to run the code available on their Github page and the documentation was unclear.

3. Is there other literature also uses the threshold of causal effect size to declare the significance? Why use 0.1, and is there any literature to support this choice?

We have not found literature applying this procedure in this exact context. However, as can be seen in Figure S7, the relation between causal effect estimate and p-value is similar to the relation between fold-change and p-value in differential gene expression studies (where the visualization in Figure S7 is called a "volcano plot"). In that context, it is in fact the default procedure to filter genes based on both a p-value threshold and an arbitrary fold-change threshold (typically two-fold, but there is no biological or other justification why this is the "right" choice). Taking an additional fold-change threshold in differential gene expression studies is done to exclude genes which attain low p-value, but without a meaningful biological effect (e.g. due to small variance). Similarly, in our analysis, we found genes with significant p-value, but small estimated causal effect (see Fig. S7), which we deemed unlikely to be biologically meaningful.

Conversely, in differential gene expression, high fold-change may occur with non-significant p-value (e.g. due to low expression levels), necessitating both a p-value as well as a fold-change cutoff. Similarly we observed genes with large estimated causal effect, but non-significant p-value (see Fig. S7).

Thus while our procedure (note that we apply both a Bonferroni corrected p-value cutoff and a causal effect size cutoff) does not appear to have a precedent in the MR literature, it is consistent with similar procedures in other contexts where there is no exact one-to-one relation between (estimated) biological effect size and statistical significance.

4. I think it is necessary to clarify the concept of regulatory pleiotropy and unmeasured pleiotropy. Besides the masking case, there exist instances where the IV affects the outcome without regulating gene expression.

We have made additional effort to explain the difference between regulatory and unmeasured pleiotropy in the Discussion.

As an aside, note that for inter-genic variants, it is hard to imagine mechanisms other than regulating gene expression by which such variants could affect phenotypes, because protein translation happens outside the nucleus. Hence the main (only?) line of communication between an inter-genic variant and protein levels and downstream phenotypes is through the regulation of mRNAs exported from the nucleus.

That said, there exists no data measuring all possible effects on gene expression in all possible cell types or tissues throughout an individual's lifetime, making this point moot and indeed necessitating a clear distinction between unmeasured pleiotropy and (measured) regulatory pleiotropy.

5. I am confused about the statement: "We focused our analysis on genome-wide significant GWAS loci because the use of eQTL information to identify candidate genes is an important part of modern GWAS protocols [see e.g. Uffelmann et al., Nat Rev Methods Primers 1, 59 (2021)] and because candidate genes at many GWAS loci have been analyzed using orthogonal data, providing some form of validation for our approach. Needless to say, our approach also could be applied to loci passing less stringent thresholds or be integrated in TWAS fine-mapping methods". Indeed, identifying the causal genes by integrating the eQTL dataset and GWAS dataset is important, and the two-sample MR require us to use the independent datasets of the exposures and the outcome. It would be better to perform the MVMR analysis without excluding the non-genome-wide significant GWAS loci and even excluding the genome-wide significant GWAS loci, and then compare the results to those in the previous manuscript. The authors' response about focusing on the genome-wide significant GWAS loci to perform the MVMR analysis is not convincing to me.

We acknowledge that in (MV)MR instruments are conventionally selected based on their association with the expososure(s), but we feel there may be a misunderstanding about the scope or purpose of the real data analysis section of our manuscript. Our purpose is to illustrate the benefits and limitations of applying MVMR with sets of correlated instrumental variables at a few well-studied GWAS loci for CAD. We explicitly do not aim to discover and validate new potential causal genes by conducting a full TWAS, which would make a full paper on its own.

Our approach of first selecting GWAS loci and then mapping the causal genes within those loci, even though only meant for illustrative purposes here, is in fact common in the GWAS literature. For instance, Uffelmann *et al.* (2021) [`https://doi.org/10.1038/s43586-021-00056-9`] describe the steps of a typical GWAS and post-GWAS strategy as: *(a)* What are the associated loci? *(b)* What are the likely causal variants? *(c)* What are the epigenomic effects of the variants? *(d)* What are the target genes in the locus? *(e)* What are the affected pathways? Our approach belongs to step *(d)* which they describe as: *"One approach for identifying regulatory target genes of genetic variants is molecular quantitative trait loci (molQTLs) analysis [...] By integrating this information with GWAS results, trait-associated variants can be mapped to the genes they are likely to regulate in specific tissues"*, i.e., precisely the approach we followed in our CAD data application.

Examples where this strategy is applied in practice are easy to find. For instance, in a GWAS for CAD [Agarwal *et al.* Nature (2022), `https://doi.org/10.1038/s41588-022-01233-6`], the

authors use a strategy that includes eQTL data and MR at genome-wide significant loci: *"To systematically prioritize likely causal genes for all 279 genome-wide associations, we integrated the following eight [...] locus-based predictors of causal genes: [...] (2) genes with eQTLs in CAD-relevant tissues, [...] (6) genes encoding proteins of causal relevance to CAD per MR studies [...]"*

Similarly, non-MR based methods for predicting causal genes underlying complex traits and diseases also often start from genome-wide significant loci and use the same approach as us to define locus boundaries and candidate genes. For instance, Weeks *et al.* [Nature Genetics (2023), `https://doi.org/10.1038/s41588-023-01443-6`] define loci as follows: *"From the set of associated variants with $P < 5 \times 10^{-8}$, we designated independent lead variants from which to define loci. [...] we performed PLINK clumping using EUR individuals in the 1000 Genomes Project reference panel with a P-value threshold of 5×10^{-8} and r2 threshold of 0.1. Within each clump, we defined the variant with the most significant P value as the lead variant. [...] We then defined the locus boundaries as 500kb on either side of the lead variant and included all genes that fell within or overlapped with the locus boundaries."* Apart from incorporating eQTL results, this is essentially the same approach we used to define loci of interest and candidate genes within them.

In summary, while we acknowledge that other approaches exist and may be more common within the (MV)MR and TWAS literature, we feel that our approach is fully consistent with the GWAS literature and befits our purpose of illustrating the benefits and limitations of our method at a few well-studied loci, as opposed to discovering entirely new candidate causal genes for CAD.

6. For the causal eQTLs, the authors sampled the instrument effect sizes randomly between 0.7 and 1.0. This setting is unrealistic.

This is a fair point. We have performed additional simulations using new effect sizes for strong and weak instruments, and revised all simulation-based figures (except Fig3 (E,F), as these simulations are computationally expensive to run). For over-determined cases where we have more instruments than exposures we sampled instrument effect sizes randomly between $0.001 - 0.01$ (weak instruments) and $0.1 - 0.3$ (strong instruments). We have also updated the F-statistic simulation to include the new effect sizes in Figure S3. For the exactly determined case where we have two instruments and two exposures, we sampled the matrix $A = [a_{ij}]$ of instrument effect sizes, where a_{ij} is the direct causal effect of E_i on X_j, randomly such that (a) its determinant was greater than 0.05 and there were no weak instruments (a_{ij} between $0.1 - 0.3$) (strong instruments), and (b) its determinant was less than 0.001 and effect sizes of each instrument less than 0.001 (weak instruments).

7. I worry about the applicability of the proposed method. If the LD matrix is not invertible, which is common when using the LD matrix from the external reference data, how could we use the GMM estimator?

One of our main results is that the least-squares (LS) or "regression of regression coefficients" method *also* solves the MVMR problem with correlated instrument sets, and that even at finite sample size, there is no practical difference between the LS and GMM methods. No LD matrix inversion is required in the LS method.

Reviewer 3

I would like to thank the authors for discussing all of my comments. I think the revision offers more clarity on the strengths and weaknesses of the proposed method. I have only a couple of further minor comments from the previous discussion.

We thank the reviewer for their appreciation and constructive comments and insights throughout the review process, which have helped to greatly improve the manuscript.

1. On the reply to point 3 – I may misunderstand the following claim in the manuscript "We filtered these eQTLs by their p-values, retaining only those with p-values below 5×10^{-8} in the GWAS study." Is the GWAS study here referred to the pQTL GWAS or the CAD GWAS? I previously thought this was referred to CAD GWAS, so I raised the questions in point 3. If the authors meant to refer to pQTL study, I suggest to rephrase this sentence.

We have carefully rewritten and expanded this paragraph to avoid any confusion. In brief, genome-wide significant SNPs that are also significant eQTLs were used to define locus boundaries and candidate genes within them in an iterative procedure. The purpose of this procedure is to define sets of instruments and exposures that form a closed causal diagram for MVMR analysis, where "closed" means that no more genes (exposures) can be added to the diagram that have shared cis-eQTL associations with any eQTLs (instruments) either already in the diagram or linked to eQTLs in the diagram.

Our motivation and justification for performing this analysis at genome-wide significant loci is outlined in our response to Reviewer 2 above.

2. I appreciate the authors' effort to perform a more comprehensive simulation studies. It would be better to provide type-I error and power results besides point estimates.

As requested, we have included type-I error and power results in new Figure S9 by simulating the procedure for selecting causal genes in the CAD data application, which consists of two steps: (a) p-value below a Bonferroni corrected threshold when testing for a non-zero causal effect (note that in the simulations we only test one gene, i.e. no correction needed) and (b) estimated causal effect greater than a threshold value c_{thresh} (in absolute value). For the type-I error, we evaluated how type-I error decreases with increasing c_{thresh}; for the power analysis, we evaluated how power increases with increasing estimated effect size at a fixed $c_{thresh} = 0.1$.

3. Could the authors explain how to determine model identifiability in practice instead of theoretically? For example, is it possible to evaluate the three conditions in the data application? If not, some discussions would be helpful too.

Model identifiability is by definition a theoretical result. It asserts whether a causal effect can be identified in principle from the structure of the hypothesized causal graph and knowledge of the observational joint probability distribution of the variables in the graph. If a causal parameter is shown to be identifiable (e.g. through one or more conditional independence tests), data can be collected to estimate parameters of the joint distribution and evaluate the identifiability conditions or causal parameter expressions.

For example, in our analysis, eq. (3) expresses the causal parameters of interest in terms of the covariance matrix of the joint p.d.f. of the variables in the model. This relation is *exact*, given the hypothesized causal graph and linearity of the model. By contrast, eqs. (7) and (9) represent two of a continuous family of finite-sample estimators for the exact eq. (3).

Causal parameter identification and estimation must be distinguished because a parameter can be identifiable but not estimable in practice, e.g. due to large sampling error with realistic sample sizes, non-invertibility of finite-sample covariance matrix estimators, etc. Conversely, if a causal parameter is shown to be non-identifiable given a hypothesized causal graph and model structure, no amount of data will make it estimable without changing the underlying hypotheses (and proving identifiability under the new hypotheses). An example where the causal parameters are non-identifiable is given in Figure 1C, corresponding to the scenario where the chosen instruments tag too small a number of true causal variants. In this case, the third instrumental set condition is violated.

We have added an explanation for the non-identifiability in Figure 1C to the SI Methods in order to further clarify and emphasize the distinction between causal parameter identification and estimation.
