# Supplementary material for: Prediction of causal genes at GWAS loci with pleiotropic gene regulatory effects using sets of correlated instrumental variables
Source: PLoS Genet. 2024 Nov 11;20(11):e1011473. doi: 10.1371/journal.pgen.1011473 (PMC11581411; doi:10.1371/journal.pgen.1011473)
Supplement: Attachment — Submitted filename: reviews-v4.pdf [file pgen.1011473.s013.pdf]

Response to the Reviewer comments

We thank the editors and all three reviewers for their appreciation and constructive comments and insights throughout the review process, which have helped to greatly improve the manuscript. We are pleased that all reviewers are happy with the previous revision.

Reviewer 3

I am happy with the authors' revision. I have one comment left: The supplementary file says that when implementing MVMR-cML, the K_vec is set as 5. However, if there is no invalid IV generated in the simulation, the K_vec should start from 0 in MVMR-cML. For example, it can be set as $0:5$*. I'd suggest the authors to double check the implementation and re-run the simulation.*

We thank the reviewer for their comment and apologize for the confusion. In the implementation and simulation of MVMR-cML, we had indeed set the K_vec to be $0:5$. We have updated the Supplementary Information file where we erroneously wrote that the K_vec is set as 5 and have replaced it with "K_vec is set to a range of 0 to 5".
